# Supplementary material for: Mithramycin induces promoter reprogramming and differentiation of rhabdoid tumor
Source: EMBO Mol Med. 2020 Dec 17;13(2):e12640. doi: 10.15252/emmm.202012640 (PMC7863405; doi:10.15252/emmm.202012640)
Supplement: Supplementary file 1 — Appendix [file EMMM-13-e12640-s001.pdf]

APPENDIX TABLE OF CONTENT

Description of each Appendix Figure and Table ……………….……………………… *1*

Figure S1: Quantifying library differences across mithramycin treatment ………..… *3*

Figure S2: Lambda read depth and library complexity and normalization ………..… *4*

Figure S3: Genome-wide peak analysis for ATAC and ChIP sequencing ....… *5*

Table S1: IC50 validation screen for cell lines treated with mithramycin ……..….. *6*

Table S2: Flow cytometry values from Extended View Figure 1 …………...........… *7*

Table S3: Genes with downregulated gene expression and chromatin accessibility at 8-hours ……………..……….……………….……………….……………..……..…. *8*

Table S4: Blood Chemistry Analysis of EC8042 treated rhabdoid tumor xenografted mice ……………..………..………………..………………..………………..…..……… *10*

Table S5: Reagent Information ……………..……..………..……………….……..…. *11*

Appendix Figure S1: Quantifying library differences across mithramycin treatment with quantro.

A: Raw library sizes for each replicate. Solvent replicates in light blue; 8-hour replicates in green; 18-hour replicates in dark blue.
B: Log2 transformed library sizes after filtering for lowly expressed genes. Solvent replicates in light blue; 8-hour replicates in green; 18-hour replicates in dark blue.
C: Log2 transformed gene-expression (voom transformed) using the filtered gene set from **B**. Solvent replicates in light blue; 8-hour replicates in green; 18-hour replicates in dark blue.

Appendix Figure S2: Lambda read depth and library complexity and normalization.

A: Lambda spike-in reads that have been de-duplicated, stratified by replicate and mithramycin treatment over time that corresponds to RUV-based accessibility normalization for ATAC-seq libraries in Figure 6.
B: Estimated ATAC-seq library complexity of individual replicates up to 10x the initial library size (first data point shown), demonstrating fundamental differences in accessibility, and by extension, library complexity from mithramycin treatment, demanding spike-in normalization procedures for adequate comparisons across drug treatment as performed in Figure 6.
C: Principal component analysis of ATAC-seq libraries prior to normalization with lambda spike-in controls. Only the first 2 principal components are shown with a scree plot showing all 9 components and variance explained.
D: Principal component analysis of ATAC-seq libraries post-normalization with lambda spike-in controls using a k of 1 for RUVg.
E: Principal component analysis of ATAC-seq libraries post-normalization with lambda spike-in controls using a k of 4 for RUVg. Notably, treatment level clusters are lost using the spike-ins as control "genes" within the first 4 factors, suggesting that library complexity can be accounted for or confounding if not addressed as dominant sources of variation in the data.
F: Principal component analysis of H3K27Ac ChIP-seq data following regressing principal component 1 out of the data.

Appendix Figure S3: Genome-wide peak analysis for ATAC and ChIP sequencing.

A-C: Heatmaps and profile tracing showing ATAC-seq peaks following mithramycin treatment for all three experimental replicates. A 2kb window is centered on the TSS.

D-F: Heatmaps and profile tracing showing H3K27ac ChIP-seq peaks following mithramycin treatment for all three experimental replicates. A 2kb window is centered on the TSS.
G,H: Quantification of genes that increase or decrease H3K27ac occupancy.
I: Donut plots representing the percentage of each chromatin super state across treatment time (from 8h to 18h) that increased 2-fold (left) or decreased 2-fold (right) in chromatin accessibility.

Appendix Table S1: IC50 validation screen for cell lines treated with mithramycin.

Appendix Table S2: Flow cytometry values from Extended View Figure 1

Appendix Table S3: Genes with downregulated gene expression and chromatin accessibility at 8-hours

Appendix Table S4: Blood Chemistry Analysis of EC8042 treated rhabdoid tumor xenografted mice

Appendix Table S5: Reagent Information

Appendix Figure S1: Quantifying library differences across mithramycin treatment with quantro.

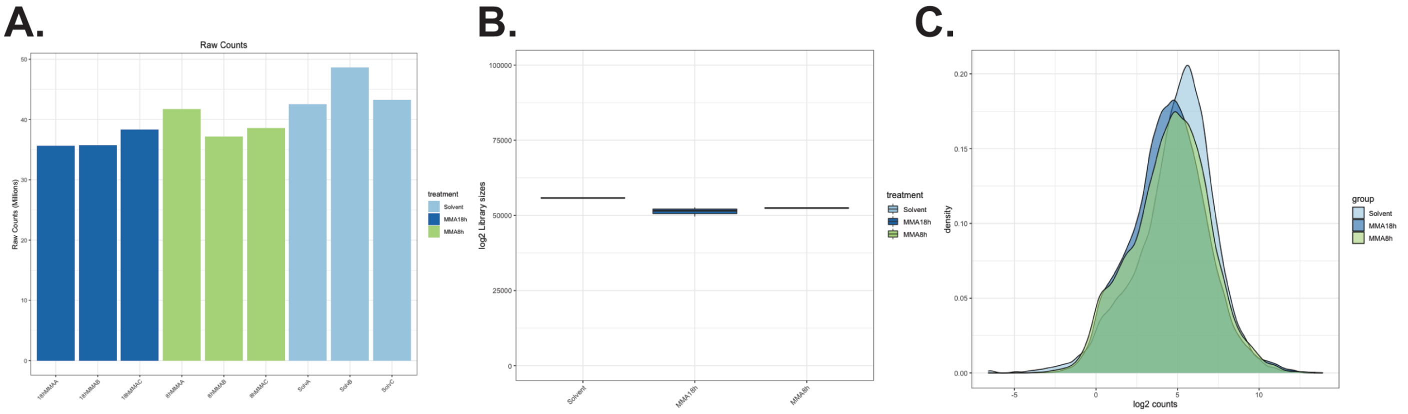

Appendix Figure S2: Lambda read depth and library complexity and normalization.

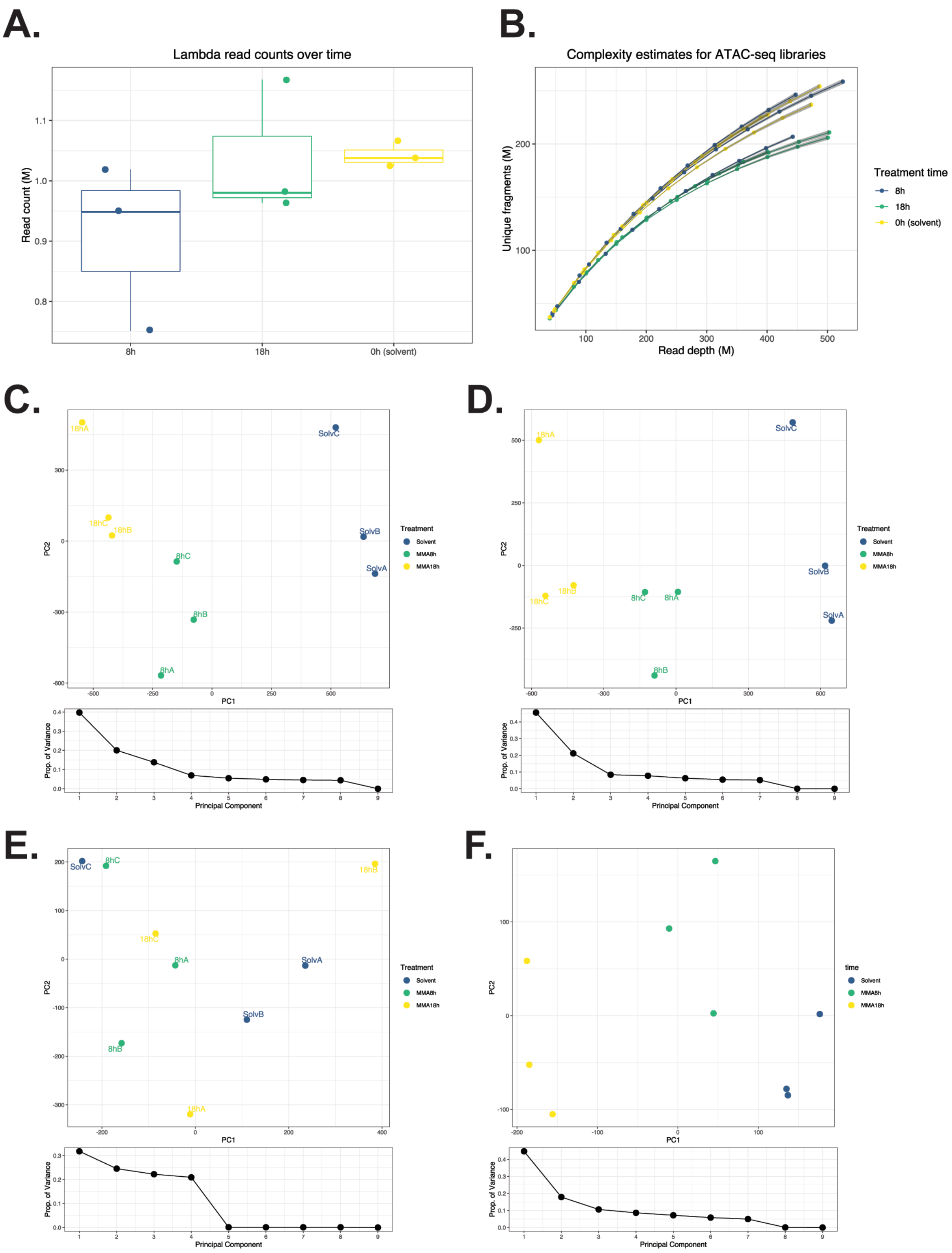

Appendix Figure S3: Genome-wide peak analysis for ATAC and ChIP sequencing.

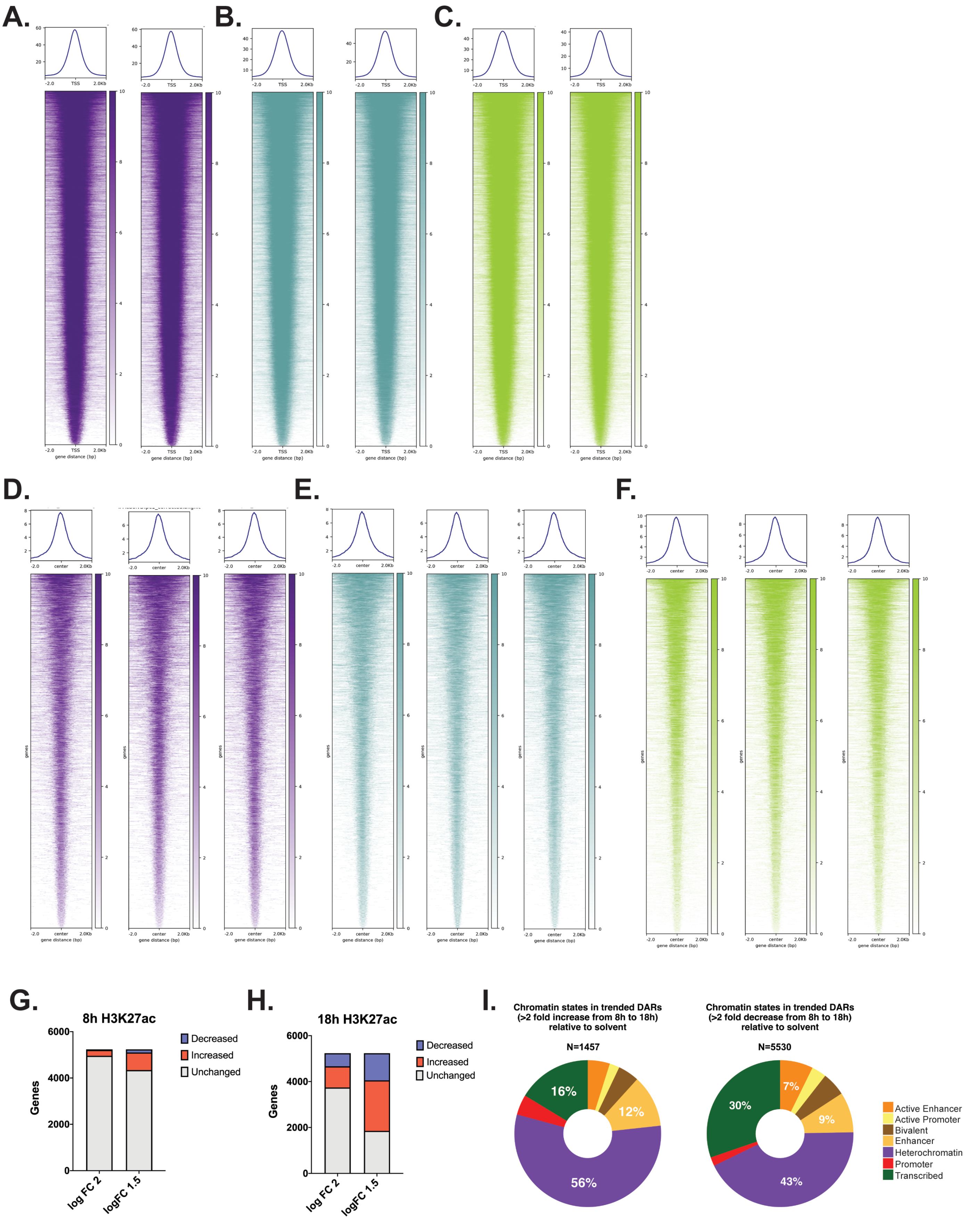

Appendix Table S1: IC50 validation screen for cell lines treated with mithramycin.

Cell Line	Histotype	IC50 (95% CI)
G402	Rhabdoid Tumor	15.0 nM (2.5-22.5)
A673	Ewing Sarcoma	15.6 nM (13-18.2)
BT12	Rhabdoid Tumor	20.0 nM (18.0-22.0)
TC32	Ewing Sarcoma	20.7 nM (18.2-23.5)
CHLA266	Rhabdoid Tumor	20.7 nM (17.7-24.0)
TTC642	Rhabdoid Tumor	23.3 nM (19.9-27.1)
BT16	Rhabdoid Tumor	25.3 nM (7.0-35.8)
RH18	Rhabdomyosarcoma	29.8 nM (26.6-33)
G401	Rhabdoid Tumor	35.8 nM (31.3-40.9)
MDA-MB-231	Breast Cancer	43.4 nM (36.7-50.8)
A204	Rhabdoid Tumor	51.7 nM (30.4-67.6)
RD	Rhabdomyosarcoma	62.7 nM (57-68.9)
MCF7	Breast Cancer	69.8 nM (60.5-80.1)
U2OS	Osteosarcoma	203.7 nM (157.4-361.1)
SKOV3	Ovarian Cancer	>2 μM

Appendix Table S2: Flow cytometry values from Extended View Figure 1

ID	Treatment	Time Point	Replicate	Cells/Single Cells/Cell Cycle | %G1	Cells/Single Cells/Cell Cycle | %S	Cells/Single Cells/Cell Cycle | %G2
1	Solvent	18h	A	59.1	30.0	9.6
2	Solvent	18h	B	53.9	33.0	10.5
3	10nM	1h	A	40.7	43.0	13.7
4	10nM	1h	B	40.2	43.2	16.7
5	20nM	1h	A	39.9	39.7	19.8
6	20nM	1h	B	40.4	42.2	16.2
7	40nM	1h	A	37.4	44.0	17.1
8	40nM	1h	B	38.9	42.4	17.7
9	10nM	8h	A	52.7	33.6	12.1
10	10nM	8h	B	51.1	34.6	12.6
11	20nM	8h	A	53.2	31.2	13.8
12	20nM	8h	B	54.4	33.8	9.8
13	40nM	8h	A	51.7	32.0	14.2
14	40nM	8h	B	52.6	31.2	13.8
15	10nM	18h	A	63.8	20.7	10.8
16	10nM	18h	B	65.8	16.9	12.6
17	20nM	18h	A	57.7	22.2	16.3
18	20nM	18h	B	56.5	29.8	15.3
19	40nM	18h	A	43.0	33.2	22.3
20	40nM	18h	B	41.2	32.5	23.2

Appendix Table S3: Genes with downregulated gene expression and chromatin accessibility at 8-hours

AATK	BAIAP2L1	CLIP1	DOK7	GPR158	LDLRAD3
ABCC1	BAZ1B	CLMP	DOT1L	GPSM1	LEMD2
ABCC2	BCAR1	CMIP	DTX2	GRHL3	LGALS9
ABCG1	BCL9L	CNKSR3	DVL1	GSE1	LINC00473
ABHD6	BEGAIN	CNNM2	DZIP1L	H2AFY2	LIPH
ABR	BFAR	CNTLN	EDN2	HDAC7	LMCD1
ACACA	BICC1	COL15A1	EFR3B	HDAC9	LMNB1
ACACB	BPTF	COL5A1	EHMT1	HEG1	LMNB2
ACVR1	BRSK2	CORO2A	ELK3	HIVEP3	LPCAT1
ADAMTS6	BRWD3	CORO7	ELMSAN1	HMG20A	LRCH1
ADORA1	C16orf74	CRTC3	EP400	HNRNPDL	LRRC8A
AFAP1	C19orf44	CSF1	EPHB2	HS6ST1	LRRC8D
AGAP3	C19orf48	CSF1R	EPS15L1	ID3	MAMLD1
AGFG2	C21orf91	CSF2RB	ERF	IFFO2	MAMSTR
AHCTF1	C2CD2	CSGALNACT1	ETV1	IFIT3	MAP2K7
AHRR	C6orf132	CSMD1	EVA1A	IGF1R	MAP3K3
AJAP1	C9orf3	CTBP1	EXOC6B	IGFBP1	MAP3K8
ALG9	CAB39L	CTIF	EXT1	IL4R	MAVS
ALPK2	CACNA1H	CUEDC1	FAM124A	INPP5D	MCC
AMIGO2	CACNA2D3	CWF19L2	FAM19A5	INTS6	MED12L
AMOTL1	CACNG4	CXADR	FAM208A	IVNS1ABP	MED22
ANKS1A	CAMK1D	CXXC5	FAM208B	JADE2	MEG9
AP1S3	CARD10	CYP24A1	FAM20C	JDP2	MEOX1
ARHGAP24	CASKIN2	CYTH1	FAT4	JMJD4	MGAT3
ARHGEF18	CASZ1	DAB2IP	FBXW11	KANK1	MICAL2
ARHGEF2	CBFA2T2	DBP	FCHO1	KANK4	MICALCL
ARHGEF4	CBLB	DCLK2	FCHO2	KAZN	MKNK1
ARID1A	CCDC12	DCLK3	FGD6	KCMF1	MLLT10
ARID3B	CCDC6	DDX58	FGF18	KCNB1	MPP3
ARL4C	CCDC93	DDX60L	FGF2	KDM4C	MSH2
ARMC7	CDKAL1	DENND2A	FMNL2	KIAA0513	MTM1
ARNT2	CDON	DENND3	FOXJ3	KIAA1211L	MTPAP
ARNTL	CELF1	DENND4C	FRMPD1	KIAA1522	MTSS1L
ARRB1	CEP192	DFFB	GAS7	KIF13A	MVB12B
ARSI	CEP72	DHRS3	GAS8	KIF18B	MYO1E
ASAP1	CGNL1	DHX37	GCNT2	KIF26B	NACC2
ASAP2	CHERP	DIP2C	GLIS2	KIF7	NAT10
ASCC1	CHKA	DIS3L2	GMPS	KIT	NAT8L
ASXL1	CHST11	DLGAP4	GNA14	KRT80	NBEA
ASXL2	CHST15	DNAH17	GNB1L	KSR1	NCOA7
ATF4	CHST3	DNAJC17	GNB3	LARP1	NCOR1
ATXN2	CIT	DNAJC5	GPATCH1	LATS2	NDST1
BAIAP2	CLASP1	DOCK5	GPR157	LDLR	NEK6

Appendix Table S3: Genes with downregulated gene expression and chromatin accessibility at 8-hours

NELFA	PPP1R9B	RNF213	SNAPC4	TNKS	ZFX
NF1	PPP2R2C	RPS6KA5	SNX30	TNRC6B	ZMYM2
NF2	PPTC7	RPTOR	SOGA1	TNS3	ZMYND8
NFAT5	PRDM16	RTKN	SORBS2	TOM1	ZNF341
NFATC2	PRKAG2	RTN4IP1	SOST	TOM1L2	ZNF346
NFIC	PRKCH	RUNX3	SPATA5	TOX2	ZNF423
NGLY1	PRKCZ	SAFB	SPECC1L	TP63	ZNF532
NIM1K	PRR5	SAMD12	SPIRE2	TPPP	ZNF568
NINL	PRRC2B	SAMD4A	SPNS2	TRAF3	ZNF608
NOL9	PSEN1	SBNO2	SPTLC3	TRAPPC12	ZNF609
NOTCH1	PTEN	SCARA5	SRCAP	TRERF1	ZNF628
NR2C2	PTPN1	SEC14L1	SREBF1	TRIM37	ZNF775
NRDE2	PTPN4	SEC14L5	SSBP3	TRIM62	ZNF787
NRF1	PWWP2B	SEMA3A	ST3GAL2	TRPM6	ZNF831
NRP1	RAB35	SEMA3C	ST3GAL4	TRPS1	
NUAK2	RAB3D	SEMA5A	ST7	TRPV3	
NUP155	RAB3GAP2	SEMA6B	STARD9	TSNARE1	
NXN	RABEP1	SETD2	STX1A	TTC23	
OSBPL5	RABGAP1	SETDB1	SUDS3	TTYH2	
PAPPA	RAD18	SETX	SYNJ2	TXK	
PARD3	RAD52	SFXN5	SYT12	UBE2R2	
PARN	RAD54B	SH2B2	TAB1	UBOX5	
PATZ1	RAI1	SH2D4A	TACC2	UBXN7	
PBX4	RALGPS1	SH3BP4	TANGO6	UNC5C	
PCDH9	RAP1GAP2	SH3PXD2A	TAOK3	USP12	
PCGF3	RAPGEF1	SH3RF2	TBC1D12	USP3	
PDGFRB	RAPGEF3	SH3TC2	TBC1D2	USP54	
PDXK	RAPGEFL1	SHANK2	TBC1D22A	VGLL4	
PDZD2	RARB	SHH	TBC1D8	VPS53	
PFKFB3	RASSF8	SHISA6	TCERG1L	VRTN	
PHACTR2	RBM33	SHROOM3	TEAD4	VSTM2L	
PIK3CB	RBM47	SIN3A	TESK2	VTI1A	
PIK3CD	RCAN1	SKI	TEX2	WDFY3	
PKD1	RCC2	SLC12A7	TFEB	WDR19	
PLCL2	RDH13	SLC19A1	THEM6	WDR7	
PLEKHA2	RGCC	SLC20A1	TIAM2	WNT9A	
PLEKHM3	RGS12	SLC29A2	TIGD5	WWC1	
PLXNA1	RHBDF2	SLC2A12	TK2	XRRA1	
PODXL	RHOF	SLC2A6	TMC5	ZBTB38	
POLR3E	RIF1	SLC38A2	TMCO4	ZBTB7B	
POU2F1	RILPL1	SLC45A4	TMEM178B	ZC3H10	
PPARGC1B	RIMS4	SLC4A7	TMEM189	ZC3H3	
PPM1H	RNF144A	SLC6A6	TMEM241	ZFAND3	
PPP1R13B	RNF182	SNAP47	TNK2	ZFHX3	

Appendix Table S4: Blood Chemistry Analysis of EC8042 treated rhabdoid tumor xenografted mice

	24-hour after treatment start		24h after treatment end	
	Vehicle	3-Day	Vehicle	3-Day
**ALB** *(g/dL)*	2.8	3.3	2.9	2.8
**ALP** *(U/L)*	43	41	43	28
**ALT** *(U/L)*	30	15	25	27
**AMY** *(U/L)*	939	860	630	671
**TBIL** *(mg/dL)*	0.3	0.3	0.3	0.4
**BUN** *(mg/dL)*	18	14	18	16
**CA++** *(mg/dL)*	9.2	9.6	9.5	10.1
**PHOS** *(mg/dL)*	5.9	6.4	7.1	6.1
**CRE** *(mg/dL)*	0.3	<0.2	<0.2	0.3
**GLU** *(mg/dL)*	147	148	152	168
**NA+** *(mmol/L)*	144	147	146	143
**K+** *(mmol/L)*	5.1	4.5	4.6	4.5
**TP** *(g/dL)*	5	5.2	4.7	4.9
**GLOB** (g/dL)	2.2	2	1.8	2.1

Appendix Table S5: Reagent Information

Chromatin Immunoprecipitation

Target	Forward	Reverse
CCND1	ACACACCATTTAGTAAAGGCCAA	AGGAATTTGGGGATTTCTCAATCAA
MYT1	ATATTGGAGCCCCAAGGGTT	CTCATTAGCAGCGGTGGCAC
GAPDH	CCTGTCCAGTTAATTTCTGACC	GCTCTGCCACCCACAGTCTGG

qPCR

Target	Forward	Reverse
SP1	TGGCAGCAGTACCAATGGC	CCAGGTAGTCCTGTCAGAACTT
SMARCC1	AGCTGTTTATCGACGGAAGGA	GCATCCGCATGAACATACTTCTT
SMARCE1	CCTGCAACACAAATGCCCAG	TTTTGGAATCGTGATACCAGAGG

Antibodies

Target	Catalog Number	Use	Dilution
Rabbit IgG	Cell Signaling Technologies #2729	Chromatin Immunoprecipitation	2 ug per ChIP
Mouse IgG	Abcam ab18394	Chromatin Immunoprecipitation	1 ug per ChIP
H3K27me3	Abcam ab6002	Chromatin Immunoprecipitation	1 ug per ChIP
H3K27ac	Active Motif #39135	Chromatin Immunoprecipitation	1 ug per ChIP
SMARCC1	Cell Signaling Technologies #71232	Chromatin Immunoprecipitation	2 ug per ChIP
SP1	EMD Millipore #07-645	Western Blot	1 to 1000, overnight 4C
EZH2	Cell Signaling Technologies #5246	Western Blot	1 to 1000, overnight 4C
SMARCB1	Cell Signaling Technologies #8745	Western Blot	1 to 1000, overnight 4C
SMARCC1	Cell Signaling Technologies #11956	Western Blot	1 to 1000, overnight 4C
SMARCE1	Cell Signaling Technologies #33360	Western Blot	1 to 1000, overnight 4C
BRD9	Cell Signaling Technologies #71232	Western Blot	1 to 1000, overnight 4C
GAPDH	Abcam ab8245	Western Blot	1 to 2000, 1-hour room temperature
H3	Cell Signaling Technologies #3638	Western Blot	1 to 5000, overnight 4C
Cleaved PARP	Cell Signaling Technologies #9546	Western Blot	1 to 1000, overnight 4C
yH2AX	Cell Signaling Technologies #9718	Western Blot	1 to 1000, overnight 4C
