# Supplementary material for: Mithramycin induces promoter reprogramming and differentiation of rhabdoid tumor
Source: EMBO Mol Med. 2020 Dec 17;13(2):e12640. doi: 10.15252/emmm.202012640 (PMC7863405; doi:10.15252/emmm.202012640)
Supplement: Supplementary file 2 — Expanded View Figures PDF [file EMMM-13-e12640-s002.pdf]

Expanded View Figures

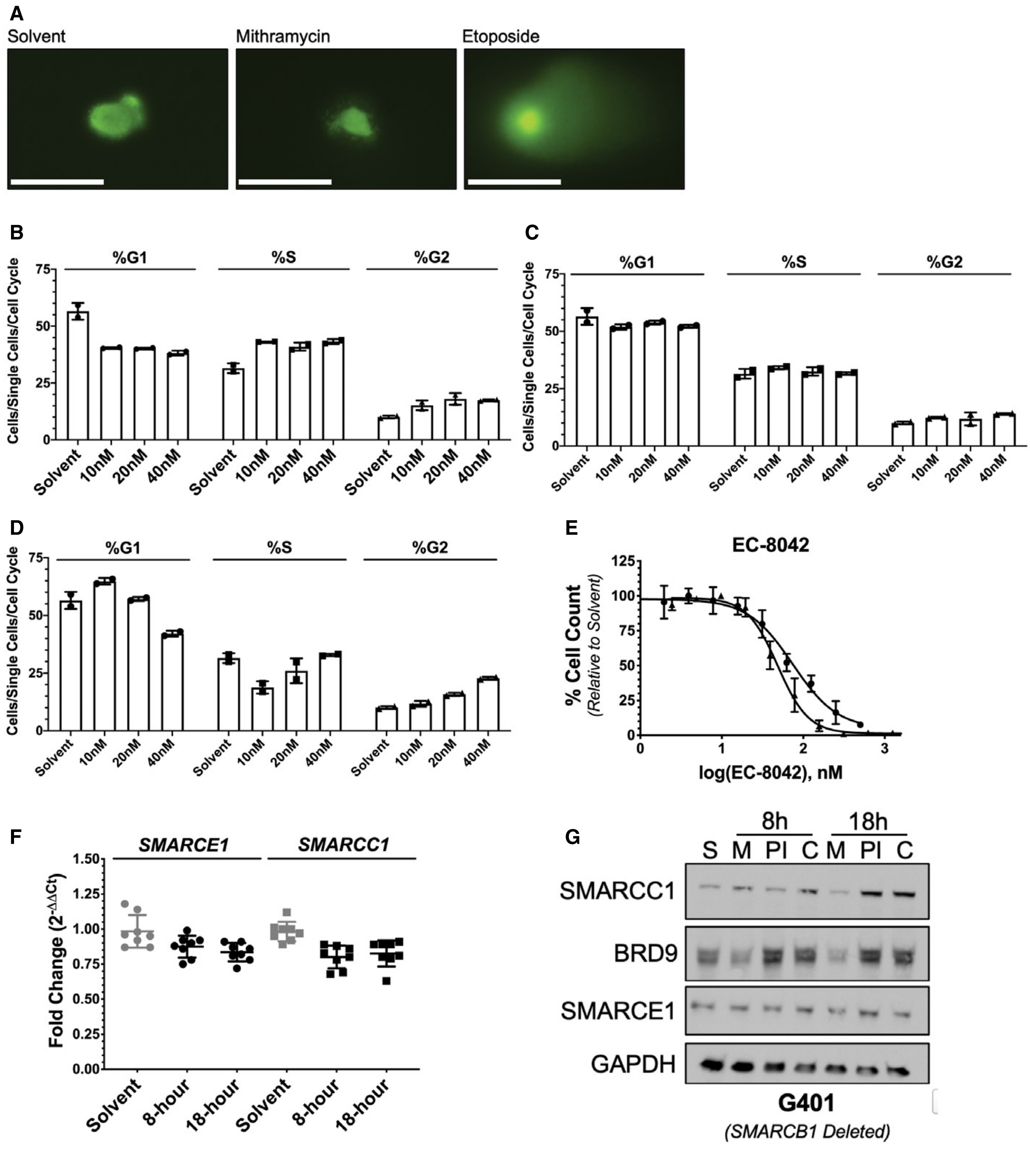

Figure EV1.

Figure EV1. Rhabdoid tumor cells are sensitive to mithramycin and EC8042, a second-generation mithramycin analogue.

A COMET assay confirmation of DNA damage from Fig 2. Solvent and 100 nM mithramycin do not lead to DNA damage after 8 h of exposure while 15 µM etoposide does. Scale bar (lower left): 50 µm.

B–D Bar graphs quantify the percent of each cell population in G1, S, or G2 following 1-h (B), 8-h (C), or 18-h (D) mithramycin exposure. Solvent was treated for 18 h. Concentrations represent 0.5× (10 nM), 1× (20 nM), and 2× (40 nM) mithramycin IC50. Values reported in Appendix Table S2. Data represent mean with standard deviation derived from three independent experiments.

E Dose–response curve of BT12 (circle) and G401 (triangle) rhabdoid tumor cells treated with EC8042. Both rhabdoid tumor cell lines are sensitive to EC8042. Data represent mean with standard deviation derived from three independent experiments.

F SMARCC1 and SMARCE1 mRNA expression does not change following 100 nM mithramycin treatment in G401 cells as measured by qPCR fold change relative to GAPDH (2^{ddCT}). Data represent mean with standard deviation derived from three independent experiments.

G Addition of the proteasome inhibitor (bortezomib) rescues the loss of protein expression following 8 and 18-h mithramycin treatment. G401 cells were treated for 8 and 18 h with solvent (S), mithramycin (M, 100 nM), bortezomib (PI, 2.5 µM), or combination (C).

Source data are available online for this figure.

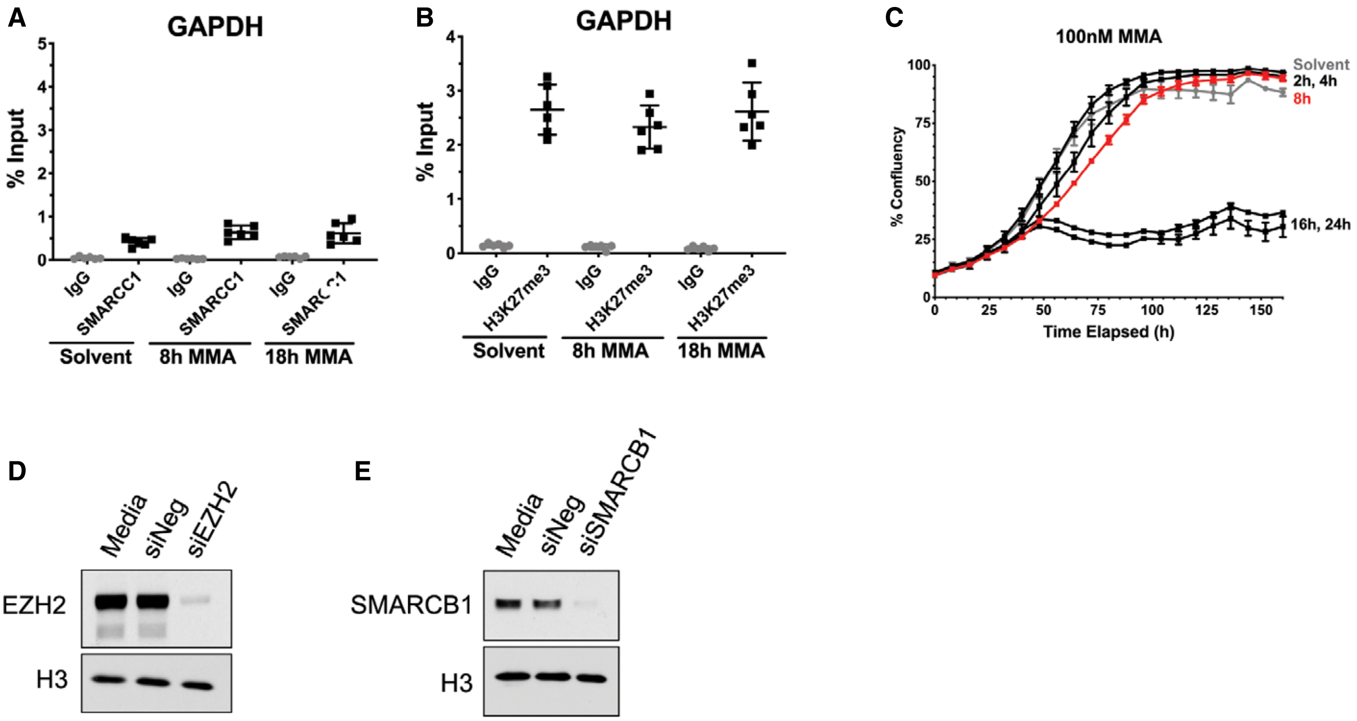

Figure EV2. Mithramycin sensitivity in rhabdoid tumor is not due to reprogramming of housekeeping genes or incomplete loss of siRNA knockdown.

A, B Chromatin immunoprecipitation of IgG, SMARCC1 (A), or H3K27me3 (B) at the control locus, *GAPDH*. Data represent mean with standard deviation derived from three independent experiments.

C Time course of 100 nM mithramycin exposure in G401 cells. Cells were treated with 100 nM MMA for the indicated times followed by a replacement of drug-free media. After 8 h (red) of mithramycin exposure, the cells have an irreversible suppression of proliferation compared to solvent control. Data from A, B represent mean with standard deviation derived from three independent experiments. Data in C are mean with standard deviation of 3 biological replicates and representative of three independent experiments.

D, E Western blot confirming efficient protein knockdown of EZH2 in BT12 rhabdoid tumor cells (D) and SMARCB1 in U2OS osteosarcoma cells (E) compared with media and siNegative controls indicative of silencing in Fig 4E and F.

Source data are available online for this figure.

© 2021 The Authors

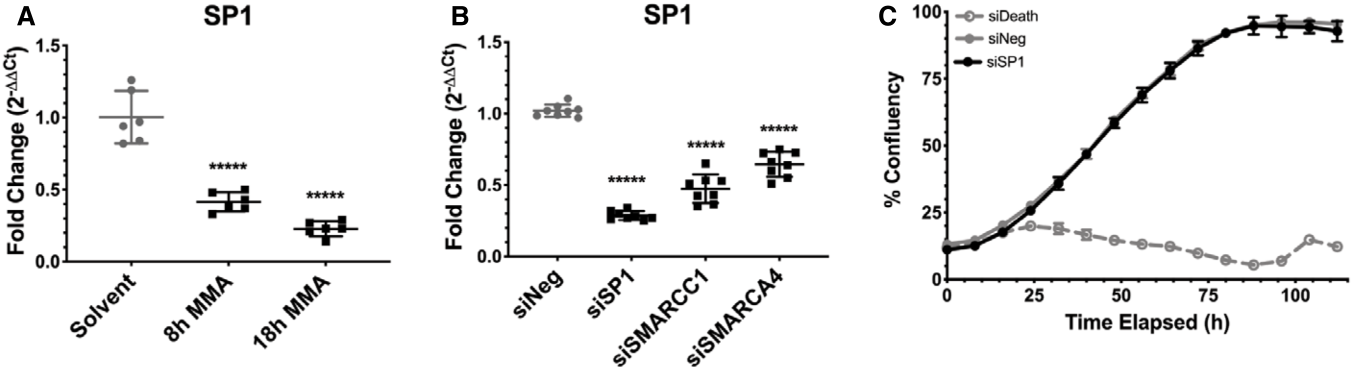

Figure EV3. SP1 expression is lost following mithramycin treatment but does not affect cell proliferation.

A SP1 mRNA expression is reduced following 100 nM mithramycin treatment as measured by qPCR fold change relative to GAPDH (2^{ddCT}) (8 h, *P* = 0.0001; 18 h, *P* = 0.0001). Data represent mean with standard deviation derived from three independent experiments. *P*-values were determined by one-way ANOVA using Dunnett test for multiple comparisons.

B SP1 expression is dependent on SWI/SNF. siRNA silencing of SMARCC1 and SMARCA4 subunits is associated with a similar loss of SP1 expression as direct silencing of SP1 as measured by qPCR relative to GAPDH (2^{ddCT}). ****P = 0.0001. Data represent mean with standard deviation derived from three independent experiments. *P*-values were determined by one-way ANOVA using Dunnett test for multiple comparisons.

C Knockdown of SP1 (black) does not affect BT12 rhabdoid tumor cell proliferation compared with a siNeg (gray, solid line) negative control as measured by live cell imaging. siDeath (gray, dotted line) is a positive control for knockdown efficiency. Data represent mean with standard deviation derived from three independent experiments.

 © 2021 The Authors

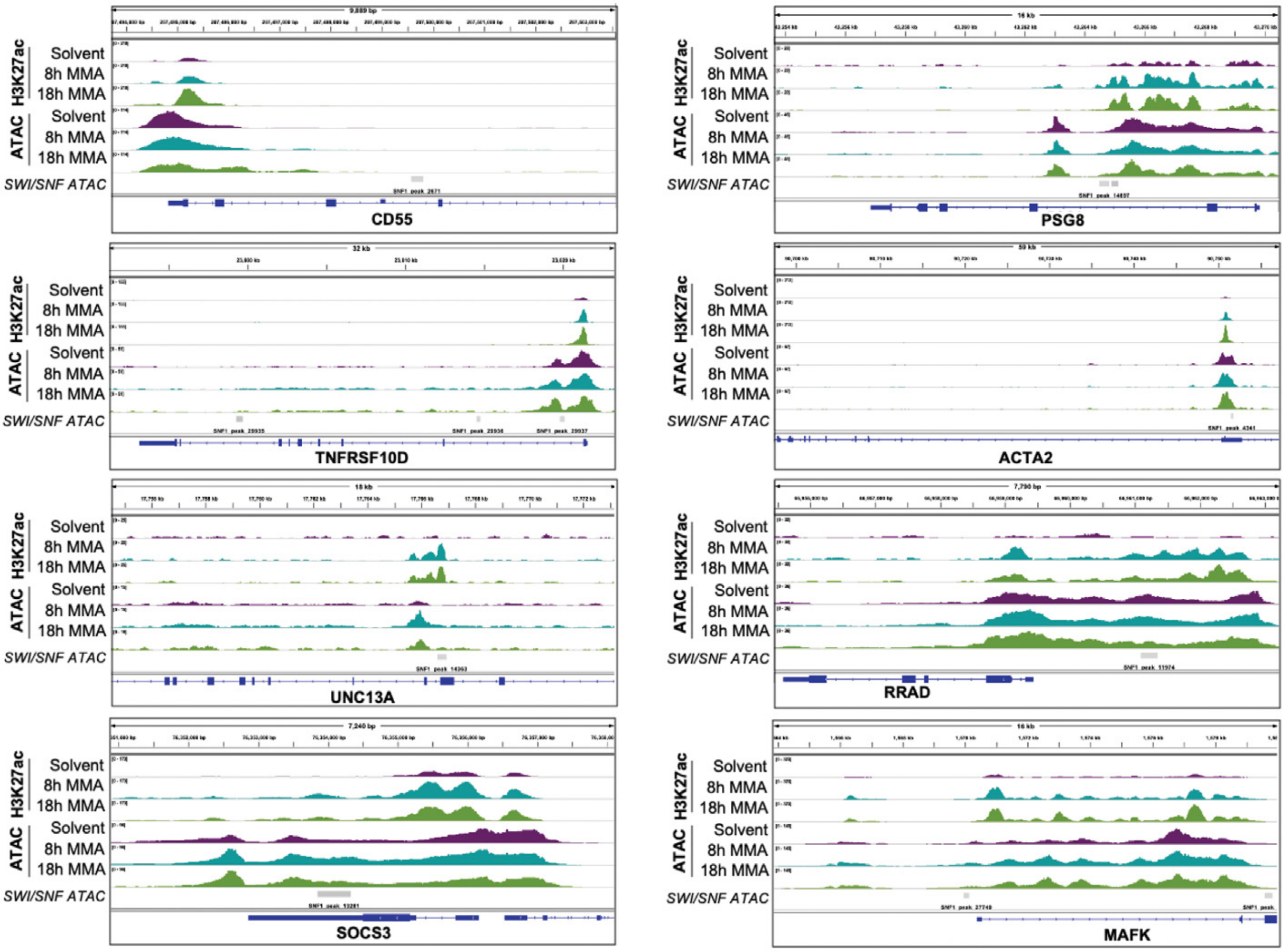

Figure EV4. IGV tracks of genes that gain accessibility following mithramycin treatment and SMARCB1 complementation.

IGV tracks of genes that increased in accessibility and H3K27ac occupancy after 18 h of 100 nM MMA exposure that correlated with previously published dataset of genes that increase in chromatin accessibility following SMARCB1 complementation in G401 rhabdoid tumor cells. Gray bars indicate peaks called from Weissmiller *et al* (2019).

Figure EV5. *In vivo* analysis of rhabdoid tumor xenografts following mithramycin and EC8042 treatment.

A Spaghetti plot showing tumor volumes of individual tumors in mice bearing G401 xenografts treated with 2.4 mg/kg of mithramycin (red) or vehicle control (gray) administered continuously intraperitoneal over 72 h. Most mice experienced a suppression or regression of tumor volume that persisted for more than 2 weeks following treatment. The shaded box indicates the duration of treatment.

B Bioluminescence imaging of G401 rhabdoid tumor xenografts correlates with caliper measurements in Fig 7A and B. Two mice per treatment group were imaged (left) and quantified in the bar graph (right). Error bars represent mean with SD. Scale bar indicates RFU intensity from (5.0e5–3.5e6, top; 1.0e6–4.0e6 bottom).

C Mice treated with EC8042 have reversible body mass loss during treatment with the 3-day infusion compared to vehicle. However, body weight recovers once treatment ends.

D Immunohistochemistry analysis of G401 xenograft tumors on 7 days (day 8) after treatment with vehicle or 3-day infusion of EC8042. 20× magnification of H&E shows osteoblasts and imbedded osteocytes in the trabecular architecture of treated xenograft tissue. Scale bar (lower left): 50 μm.

E PCA analysis of mithramycin-treated BT12 cells with normal skull. Mithramycin-treated cells cluster more with skull compared with solvent.

© 2021 The Authors

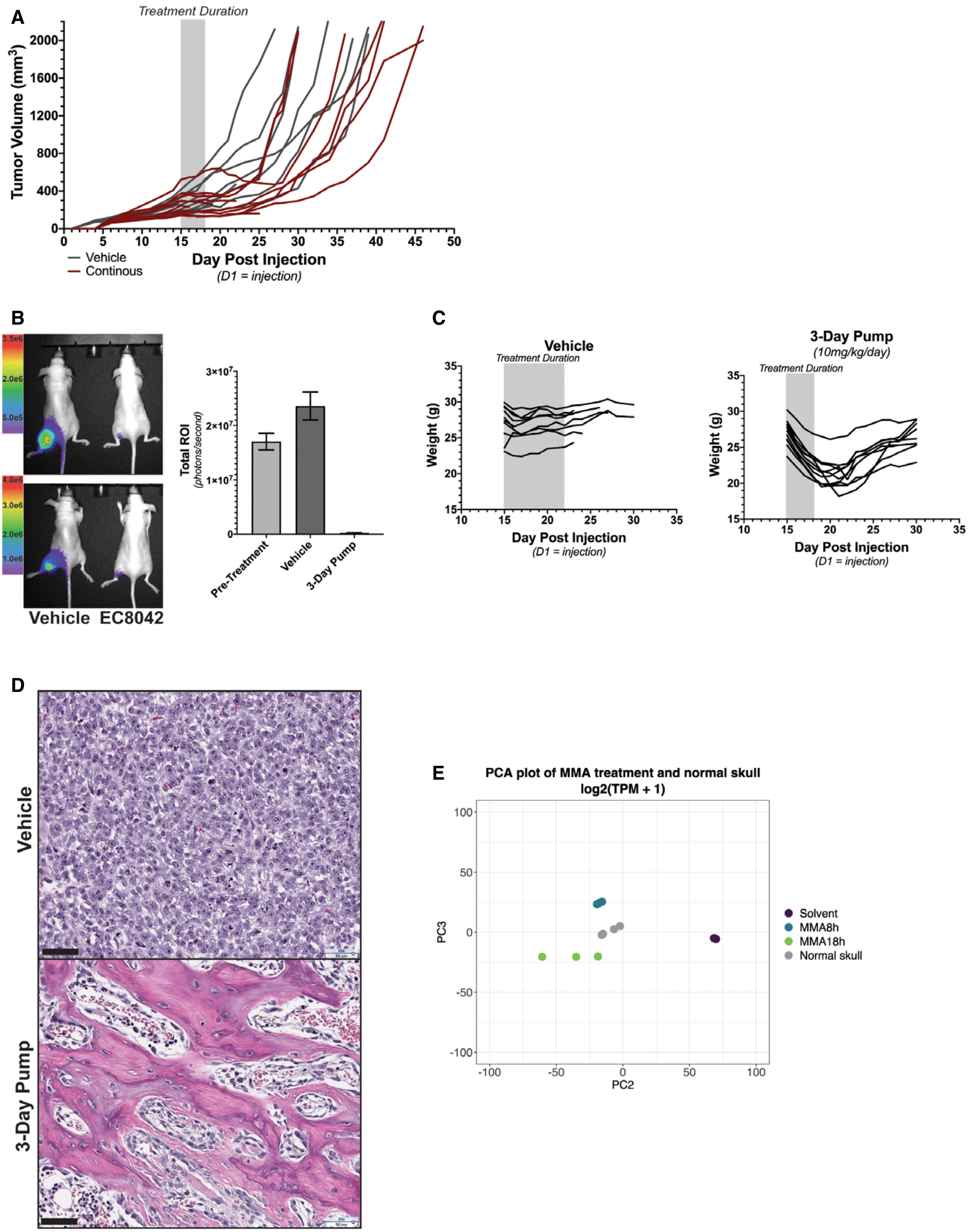

Figure EV5.

 © 2021 The Authors
