# Supplementary material for: Mithramycin induces promoter reprogramming and differentiation of rhabdoid tumor
Source: EMBO Mol Med. 2020 Dec 17;13(2):e12640. doi: 10.15252/emmm.202012640 (PMC7863405; doi:10.15252/emmm.202012640)

Source Data for Chasse 2020, Extended View Figure 1A

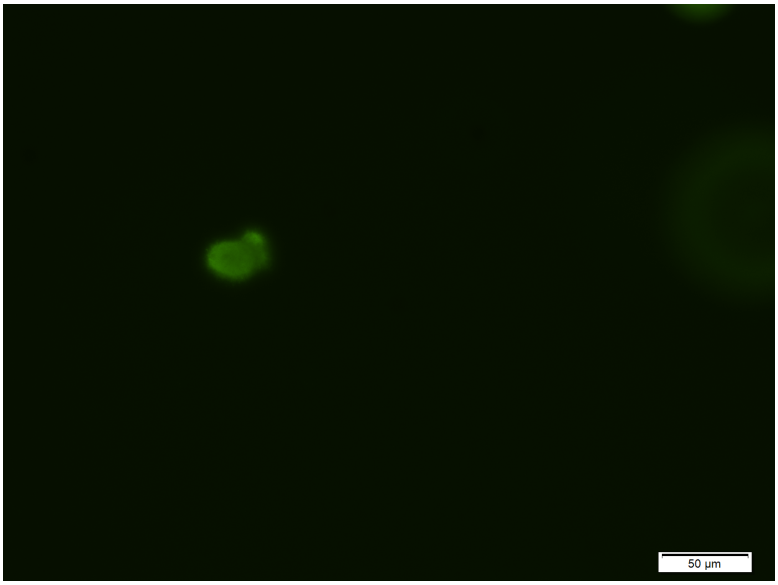

Solvent

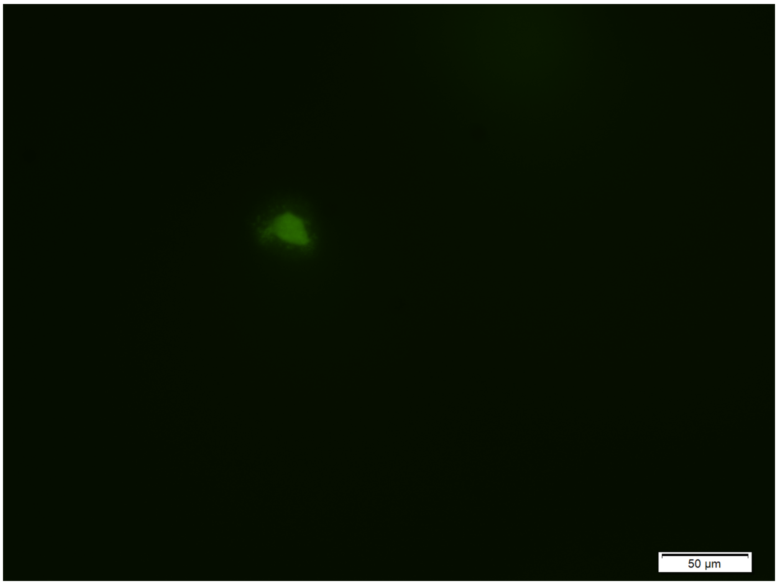

Mithramycin

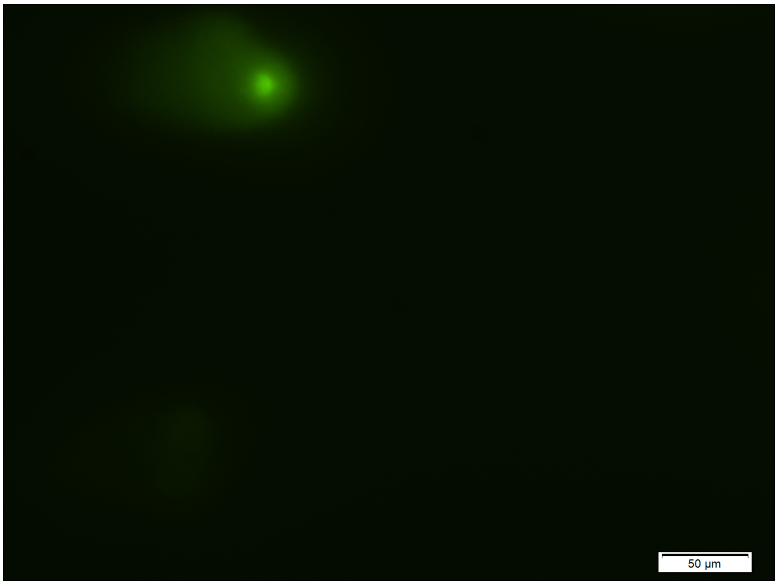

Etoposide

Source Data for Chasse 2020, Extended View Figure 1G, G401

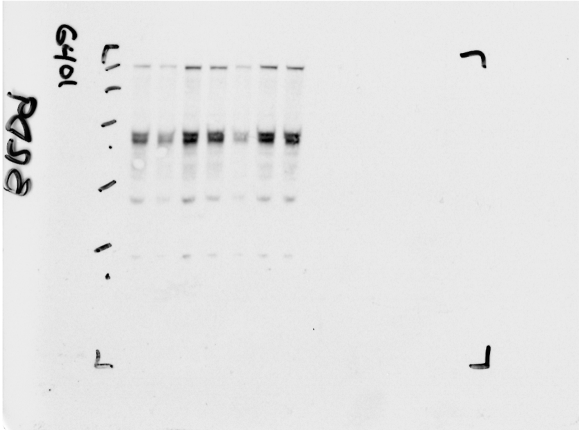

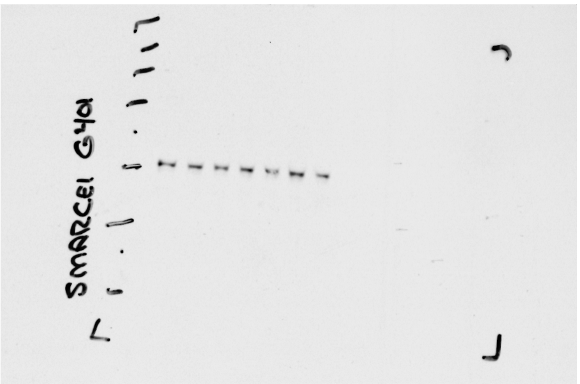

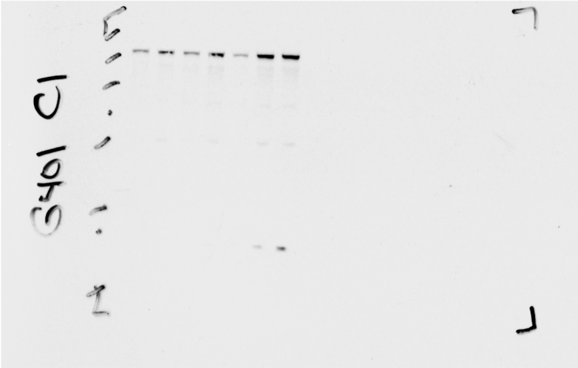

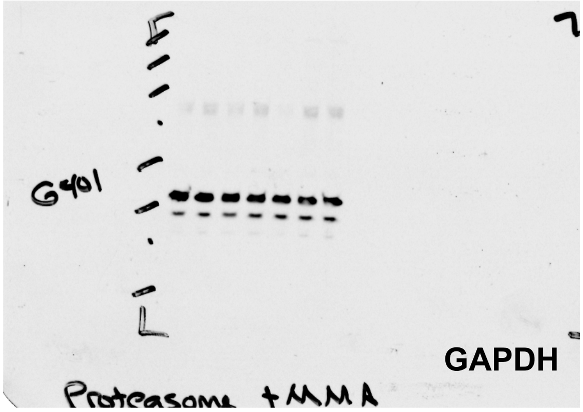

Supplement: Supplementary file 3 — Source Data for Expanded View and Appendix [file EMMM-13-e12640-s007.zip › EMM-2020-12640-V3-FigureEV1_Source_Data-sd.pdf]
