# Supplementary material for: Mithramycin induces promoter reprogramming and differentiation of rhabdoid tumor
Source: EMBO Mol Med. 2020 Dec 17;13(2):e12640. doi: 10.15252/emmm.202012640 (PMC7863405; doi:10.15252/emmm.202012640)

Source Data for Chasse 2020, Figure 2A

yH2AX BT12

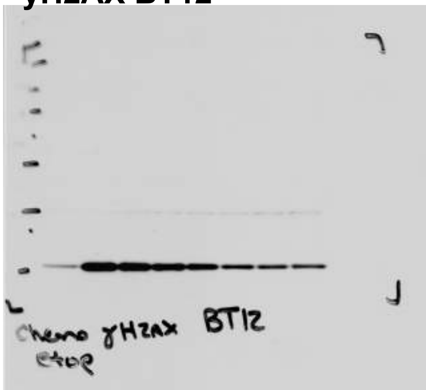

yH2AX G401

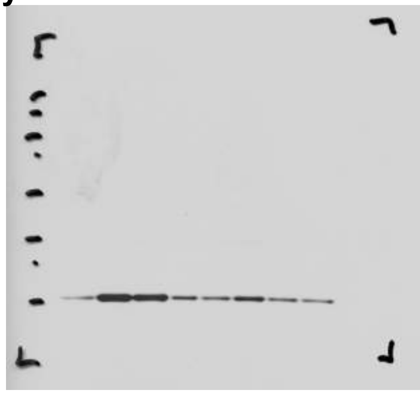

H3 BT12

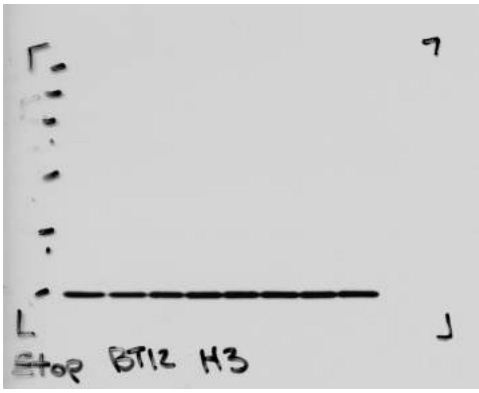

H3 G401

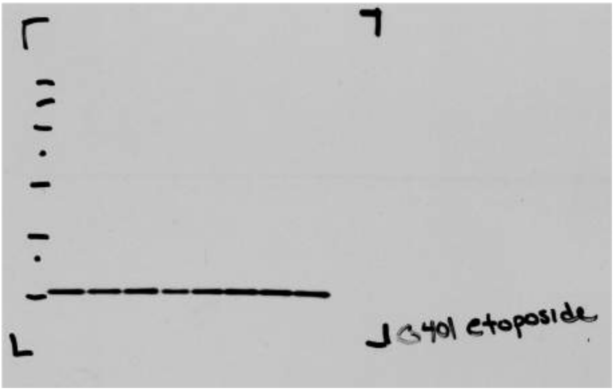

Source Data for Chasse 2020, Figure 2B

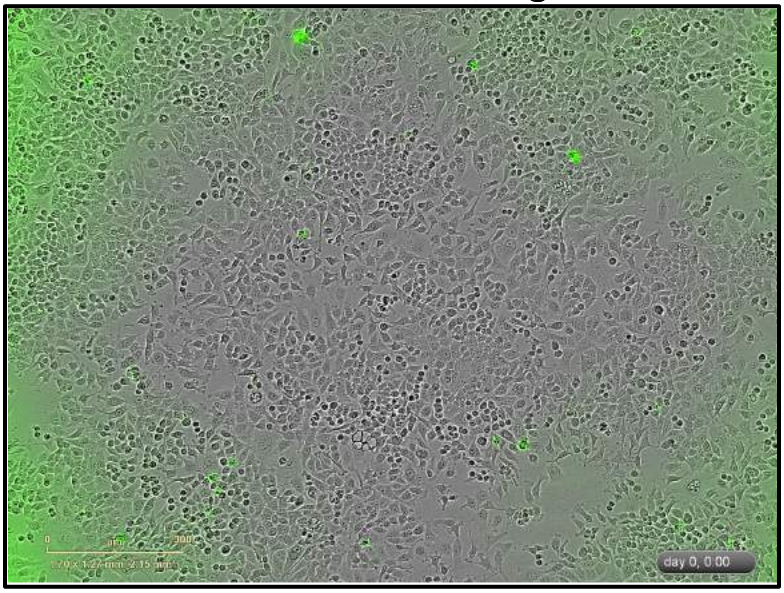

Solvent

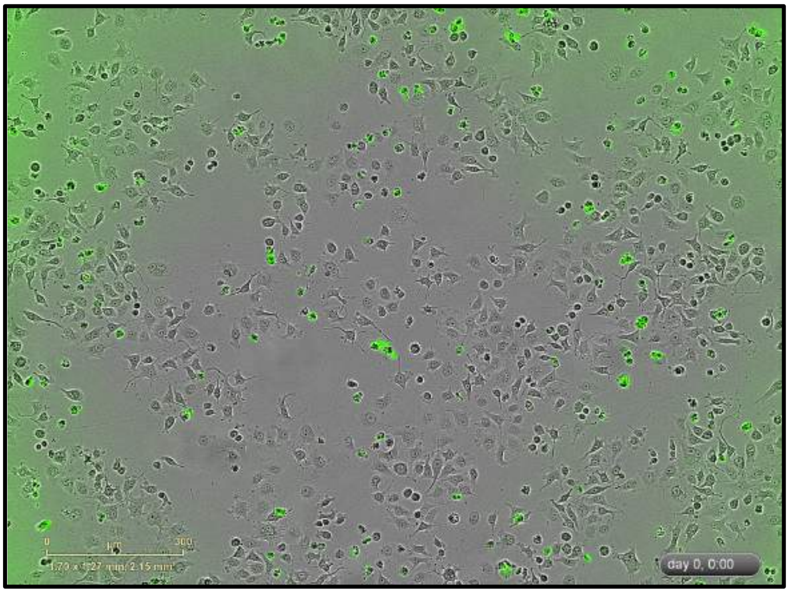

8-hours

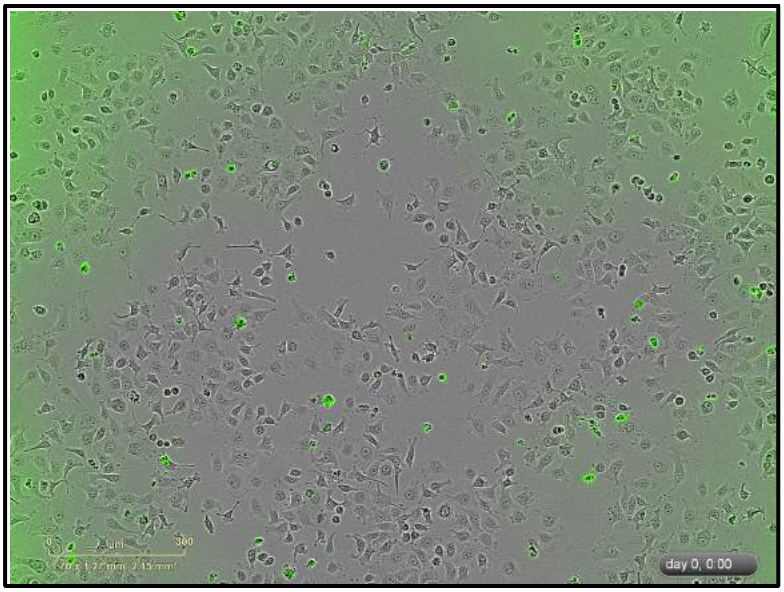

24-hours

Source Data for Chasse 2020, Figure 2C

yH2AX BT12

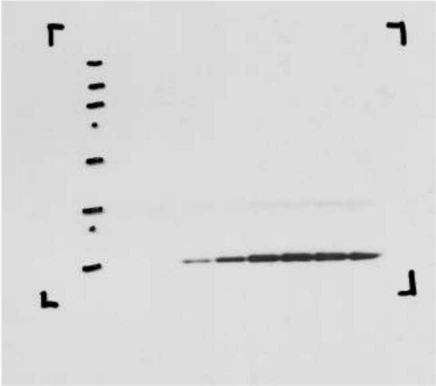

yH2AX G401

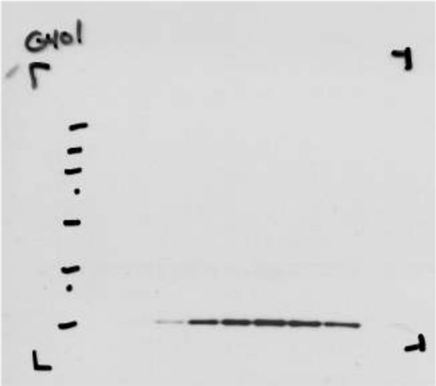

H3 BT12

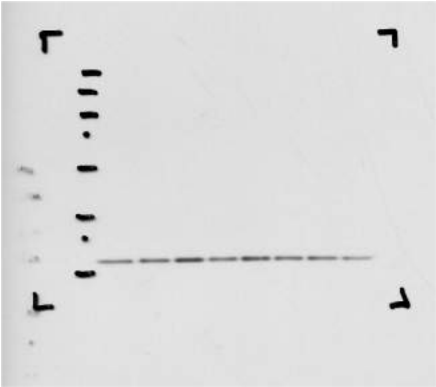

H3 G401

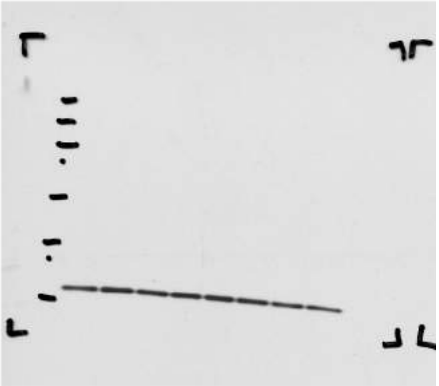

Source Data for Chasse 2020, Figure 2D

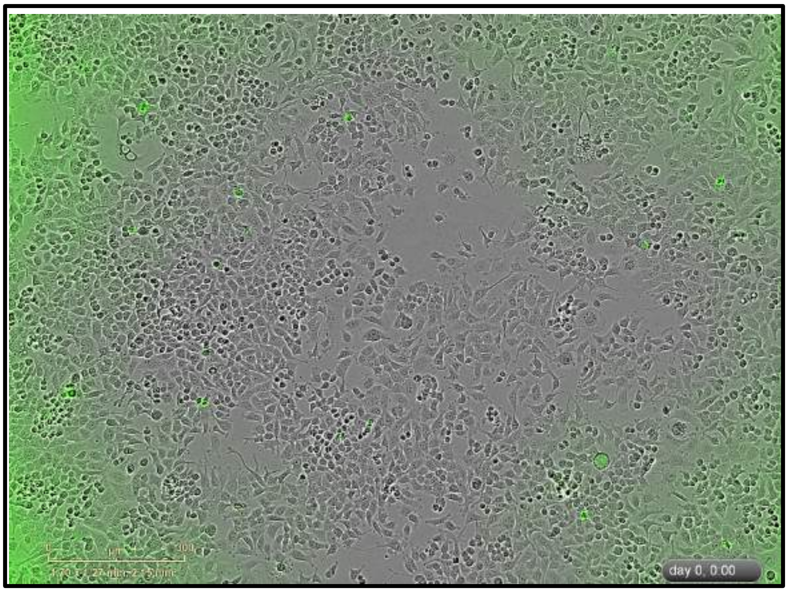

Solvent

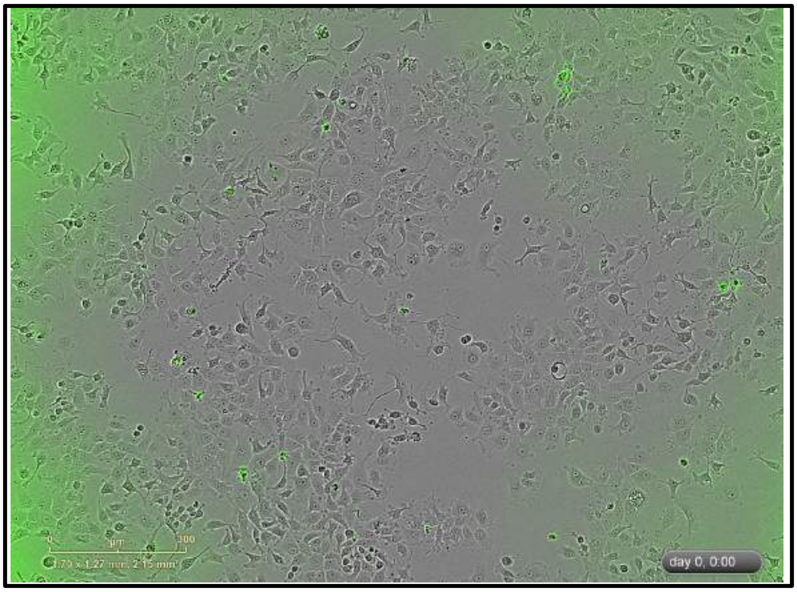

8-hours

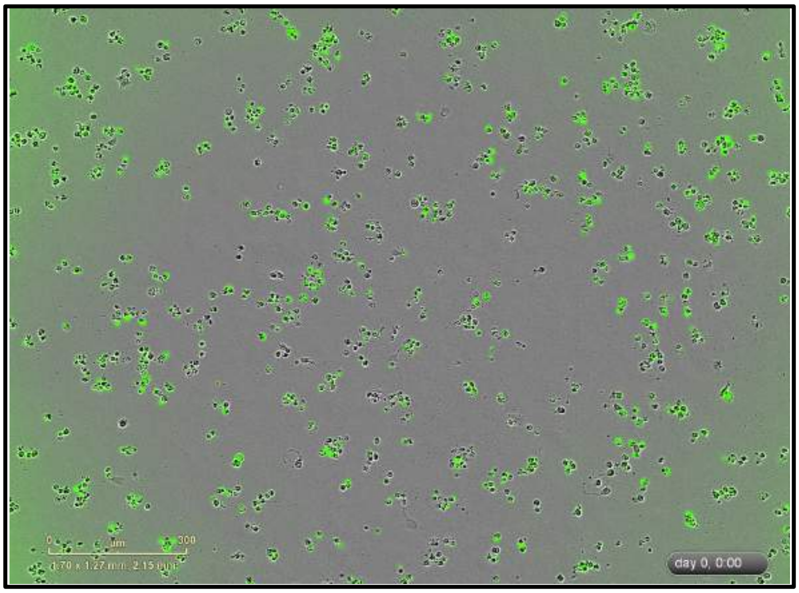

24-hours

Source Data for Chasse 2020, Figure 2E

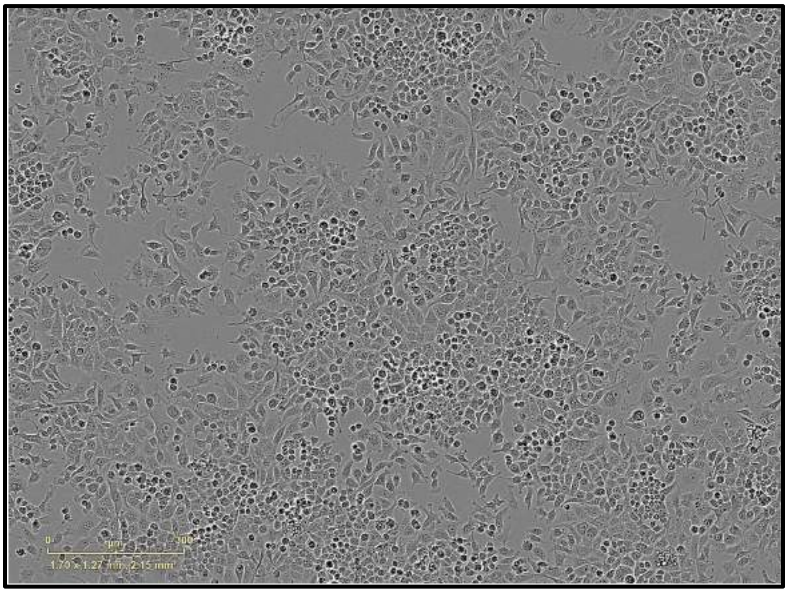

Solvent

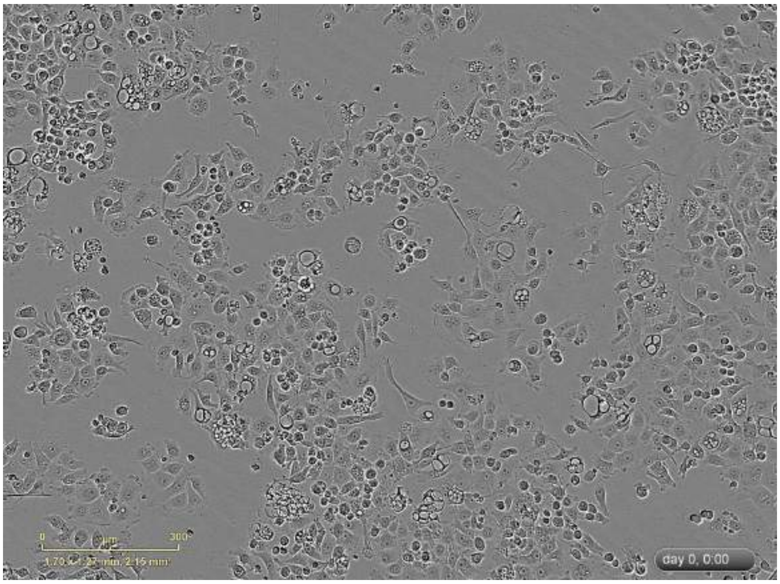

24-hours

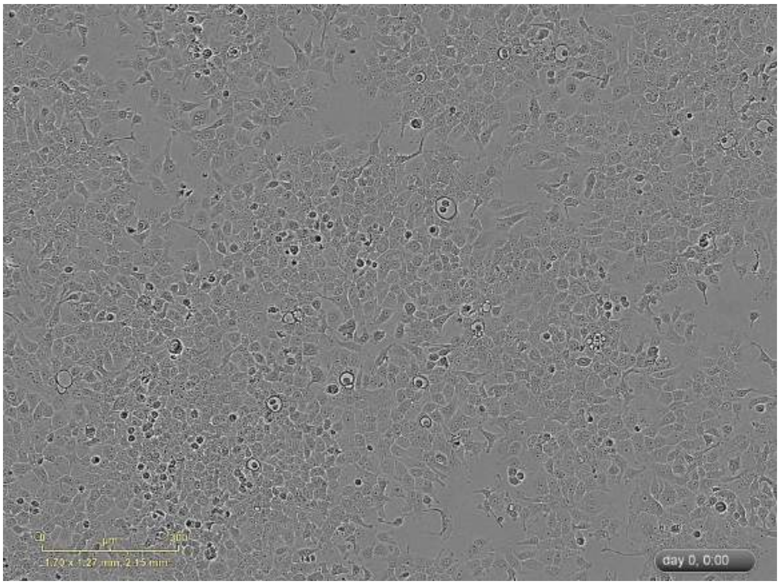

48-hours

Source Data for Chasse 2020, Figure 2F

yH2AX BT12

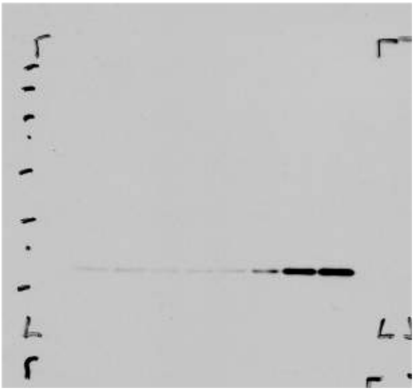

yH2AX G401

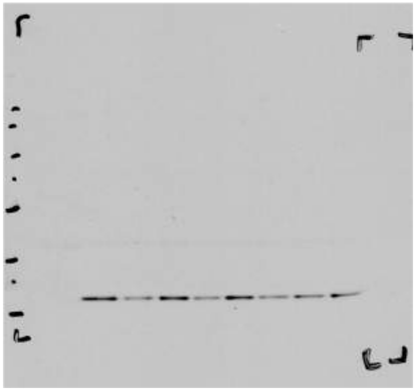

H3 BT12

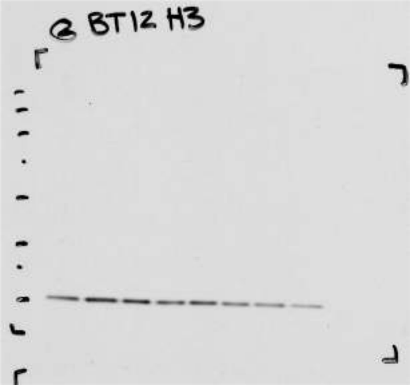

H3 G401

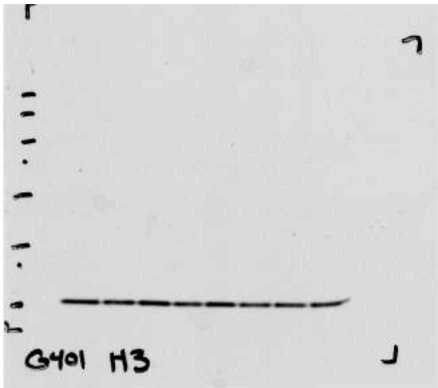

Source Data for Chasse 2020, Figure 2G

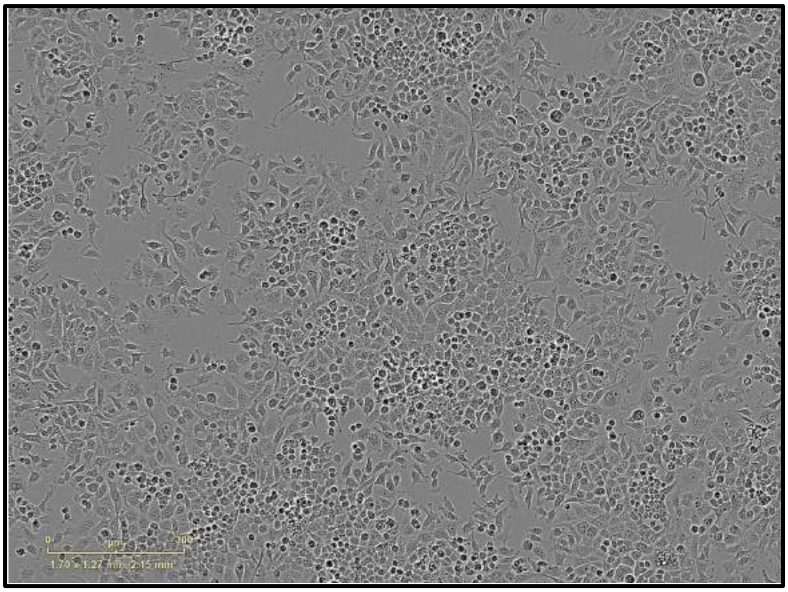

Solvent

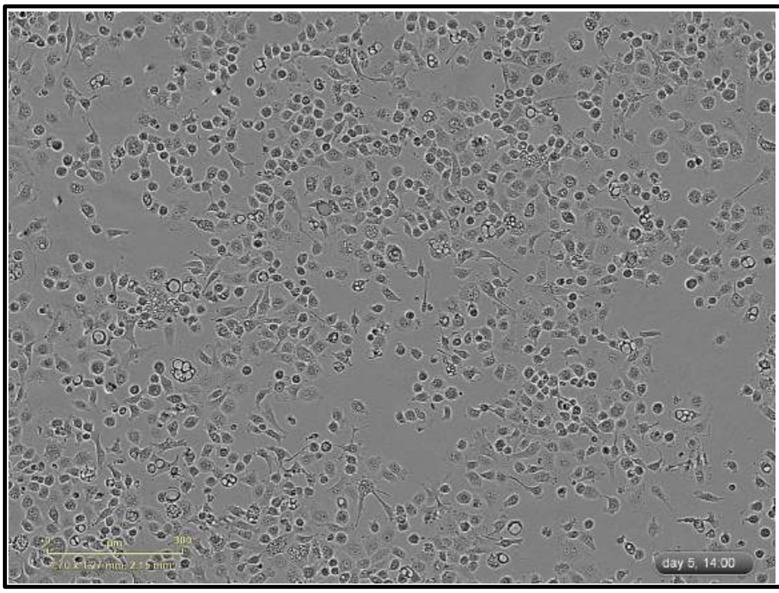

24-hours

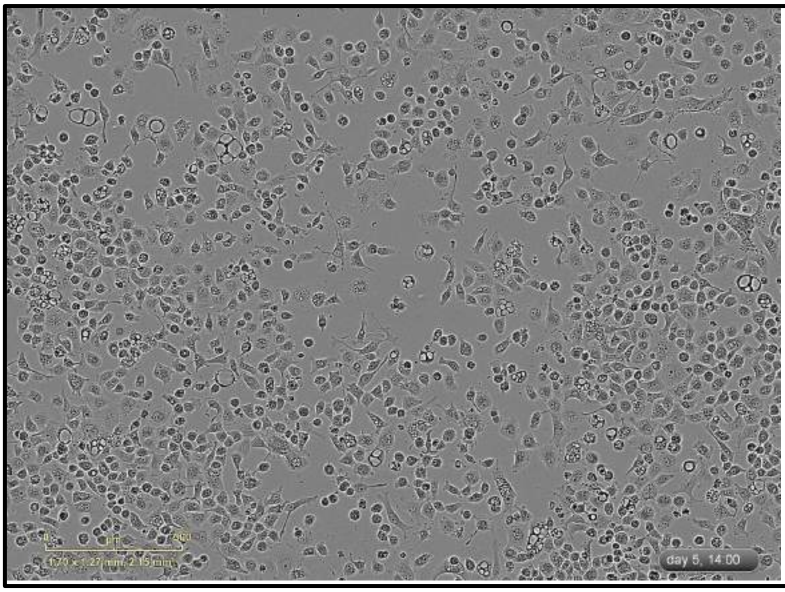

48-hours

Source Data for Chasse 2020, Figure 2H

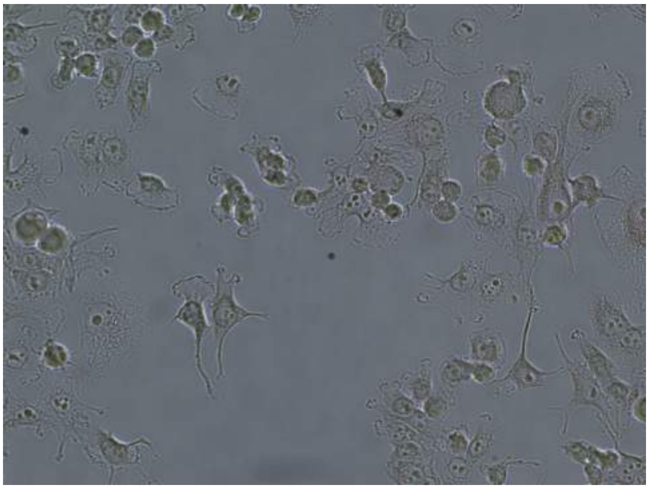

Solvent

24h 20nM Mithramycin

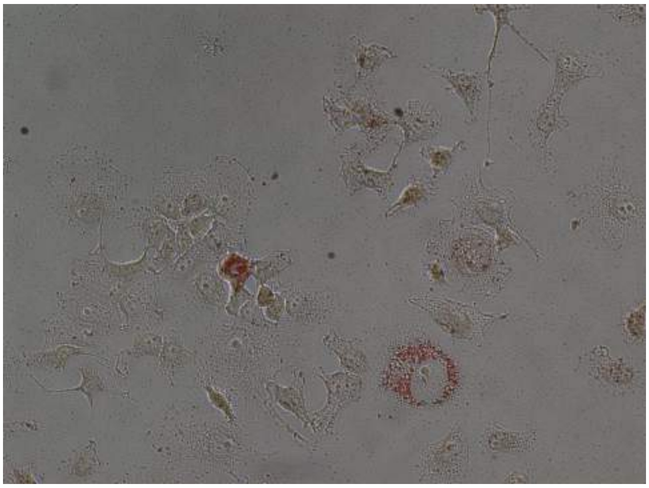

48h 20nM Mithramycin

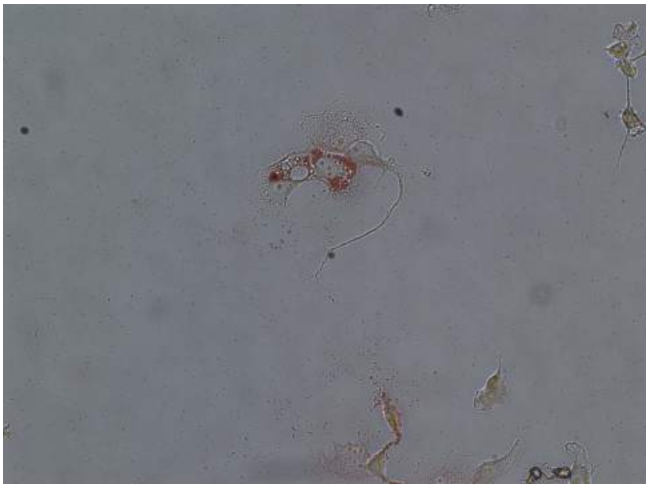

24h 75nM EC8042

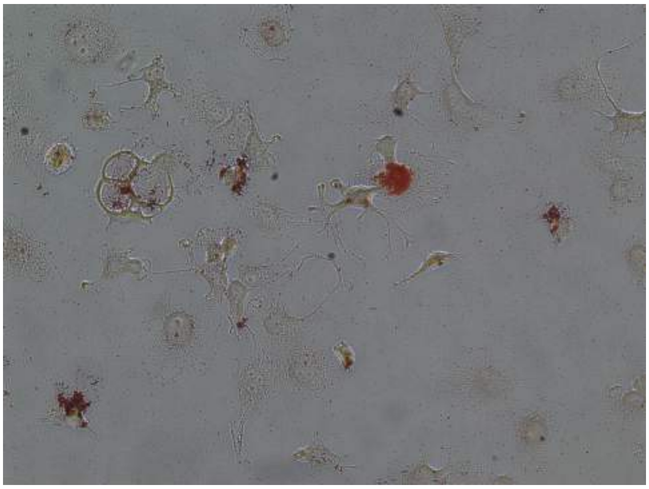

48h 75nM EC8042

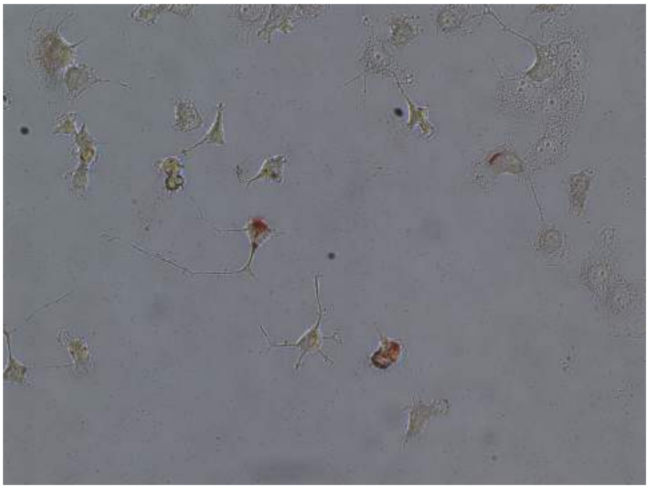

Supplement: Supplementary file 5 — Source Data for Figure 2 [file EMMM-13-e12640-s003.pdf]
