# Supplementary material for: Mithramycin induces promoter reprogramming and differentiation of rhabdoid tumor
Source: EMBO Mol Med. 2020 Dec 17;13(2):e12640. doi: 10.15252/emmm.202012640 (PMC7863405; doi:10.15252/emmm.202012640)

Source Data for Chasse 2020, Figure 3A BT12

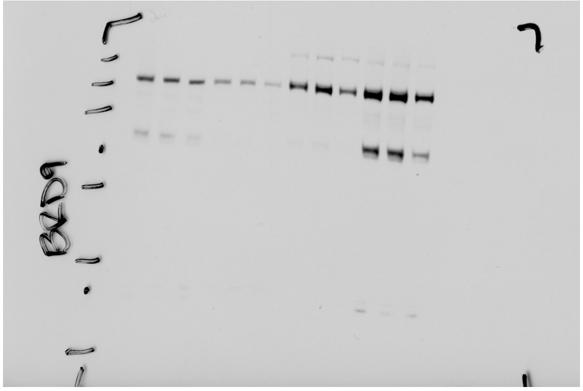

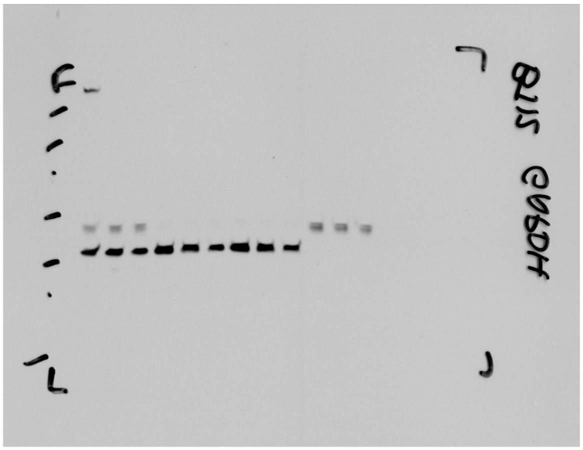

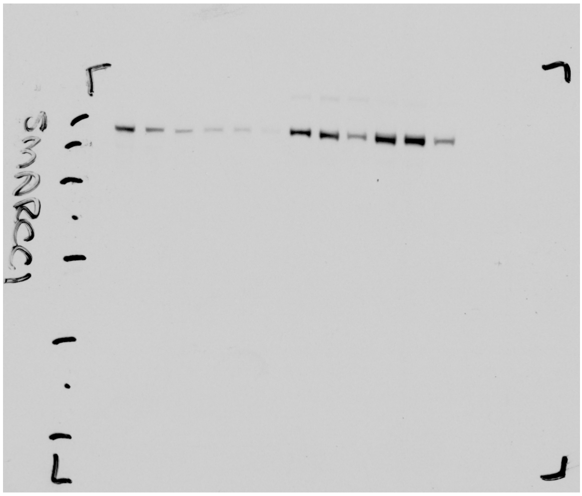

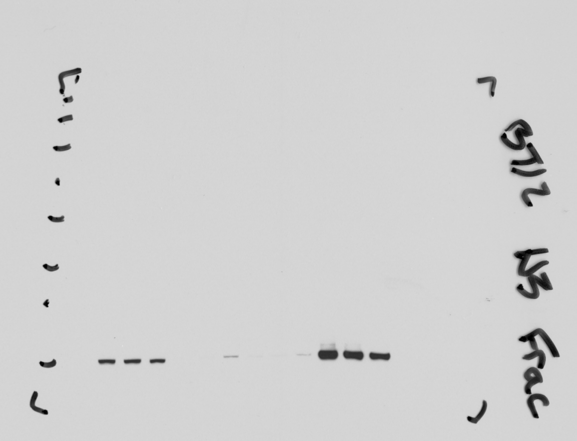

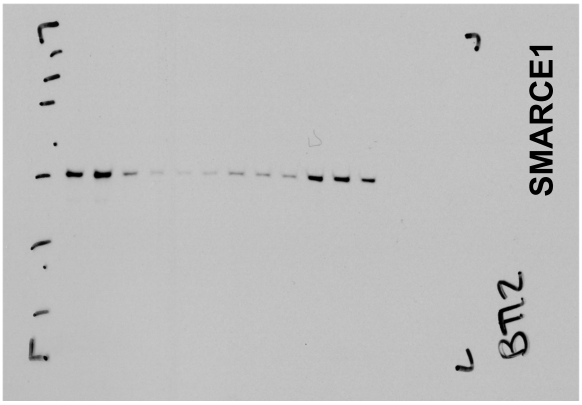

Source Data for Chasse 2020, Figure 3A U2OS

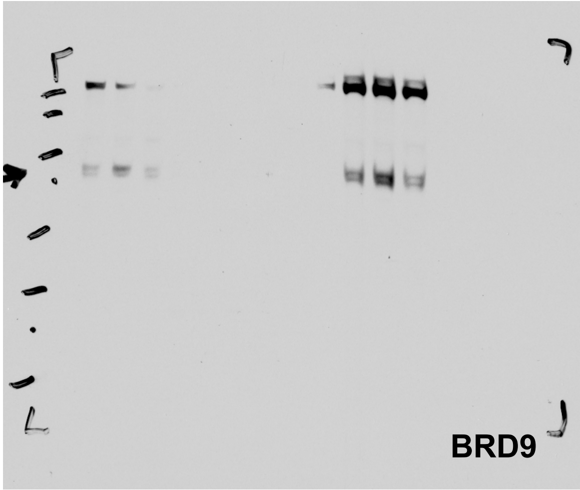

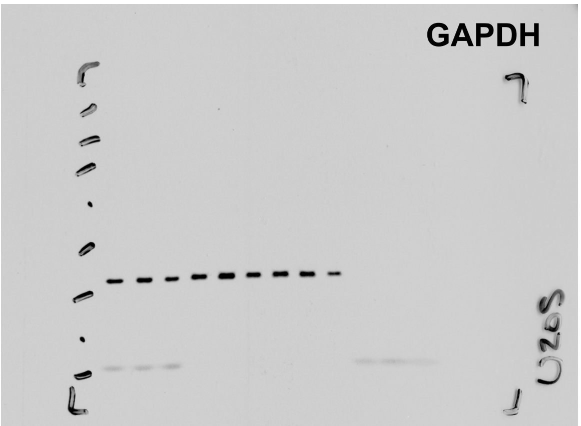

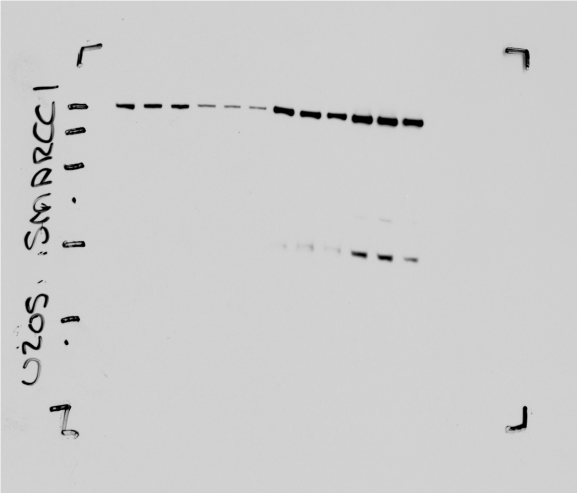

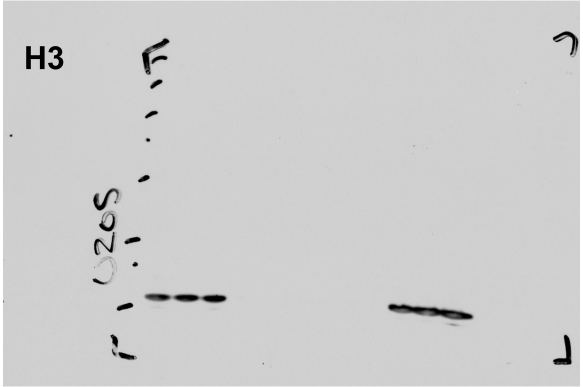

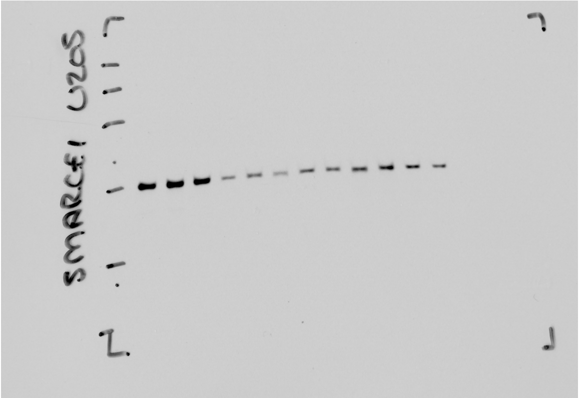

Source Data for Chasse 2020, Figure 3D BT12

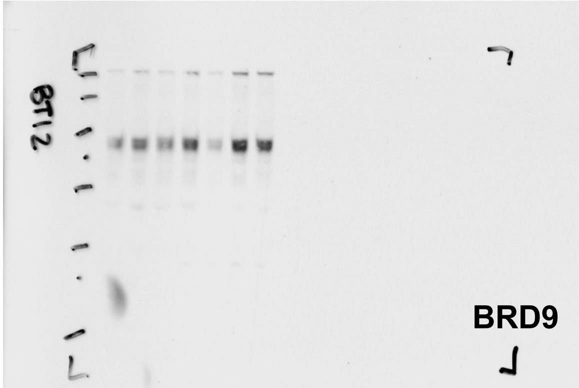

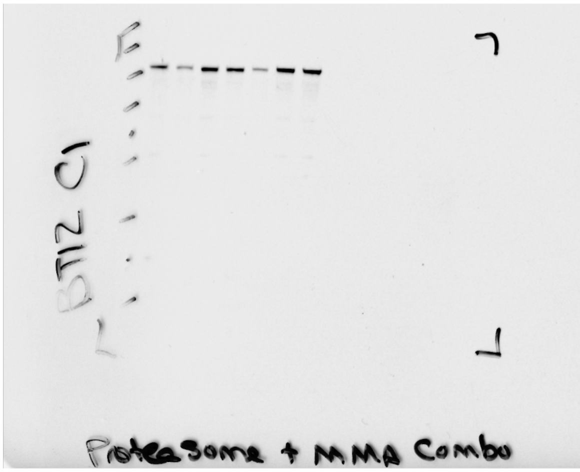

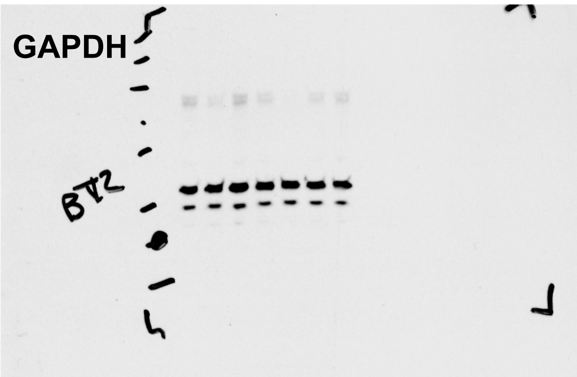

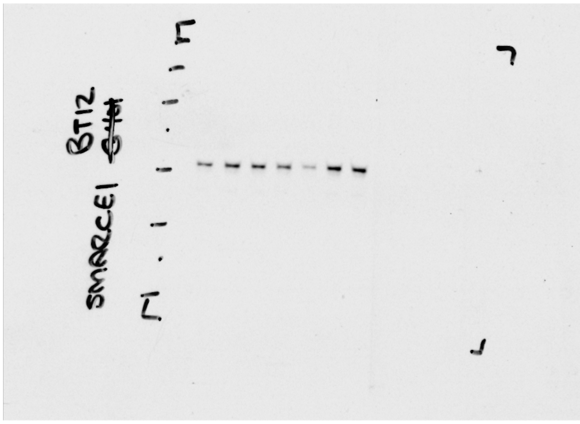

Source Data for Chasse 2020, 3G, BT12

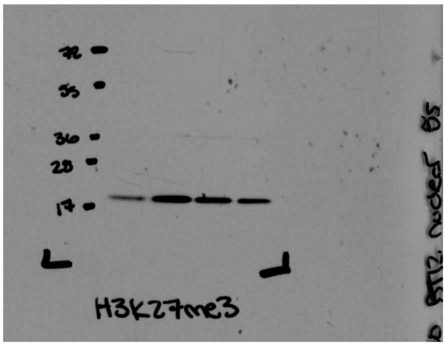

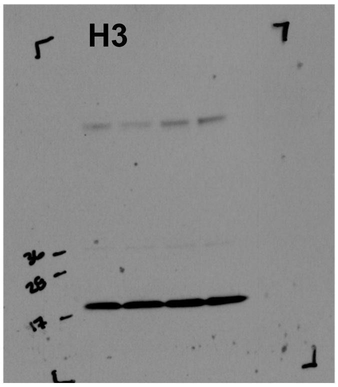

Source Data for Chasse 2020, 3H & I DOX INDUCIBLE

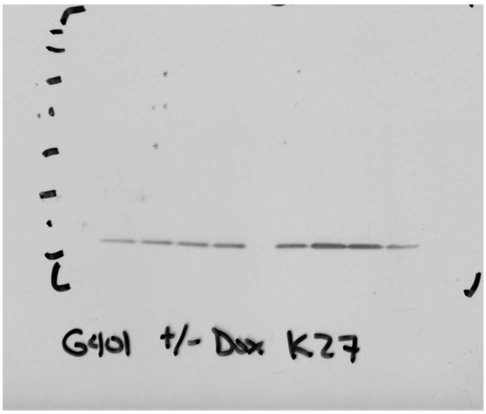

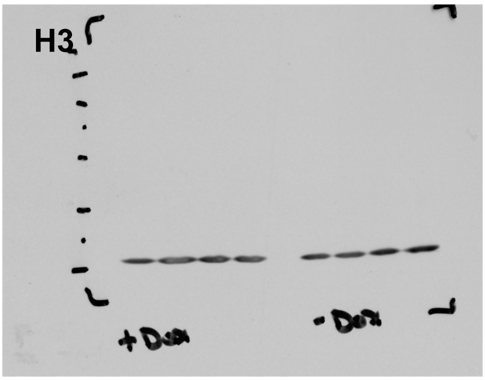

Supplement: Supplementary file 6 — Source Data for Figure 3 [file EMMM-13-e12640-s004.pdf]
