# Supplementary material for: Mithramycin induces promoter reprogramming and differentiation of rhabdoid tumor
Source: EMBO Mol Med. 2020 Dec 17;13(2):e12640. doi: 10.15252/emmm.202012640 (PMC7863405; doi:10.15252/emmm.202012640)

Source Data for Chasse 2020, 4A G401

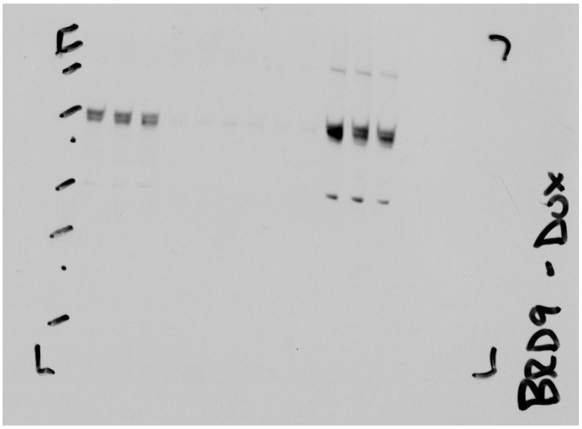

LADDER ON WRONG SIDE
SMARCE1

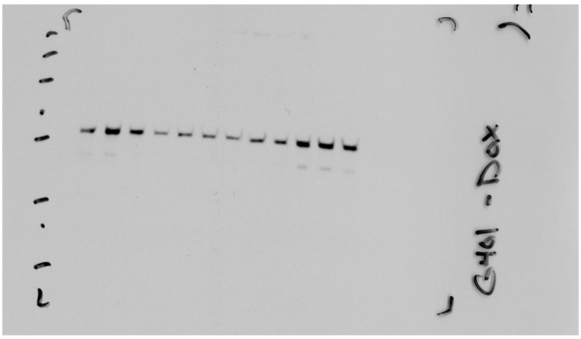

GAPDH

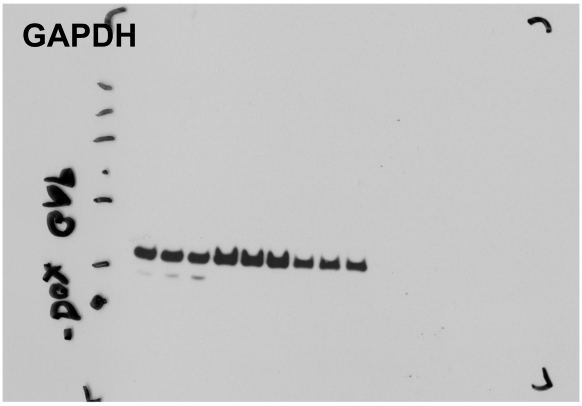

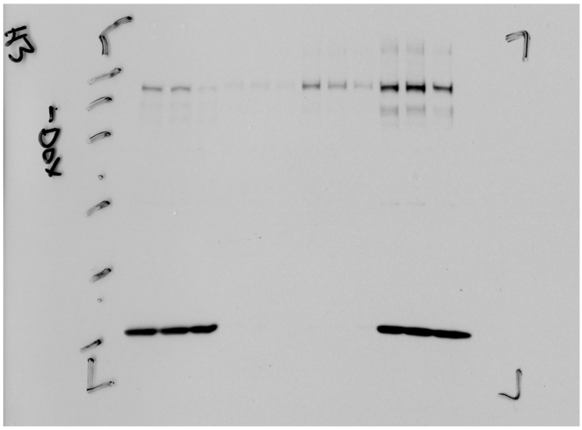

Source Data for Chasse 2020, 4B G401 + DOX

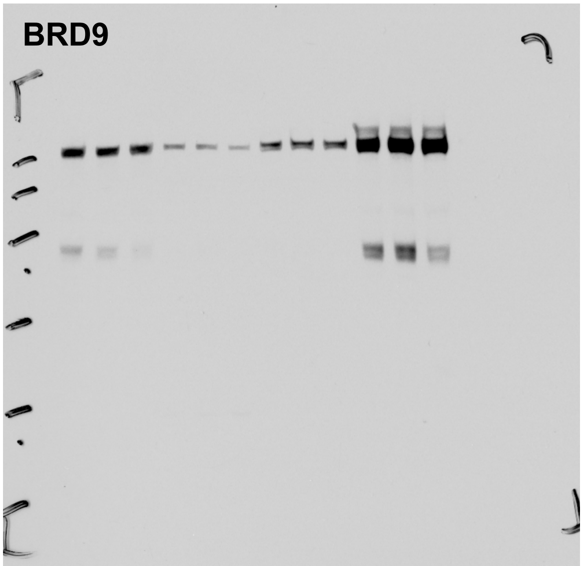

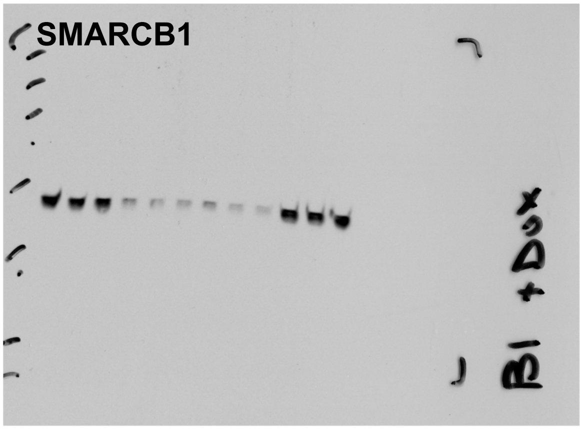

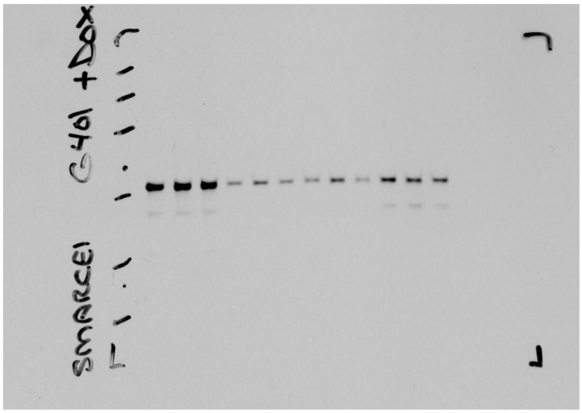

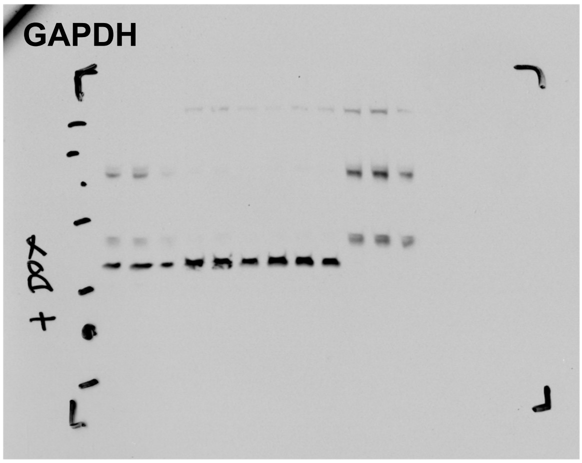

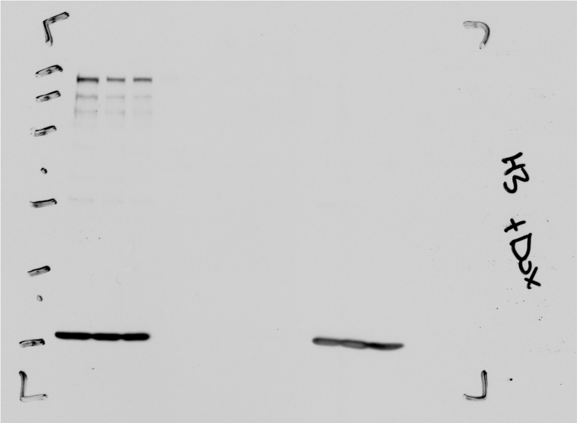

Source Data for Chasse 2020, 4D

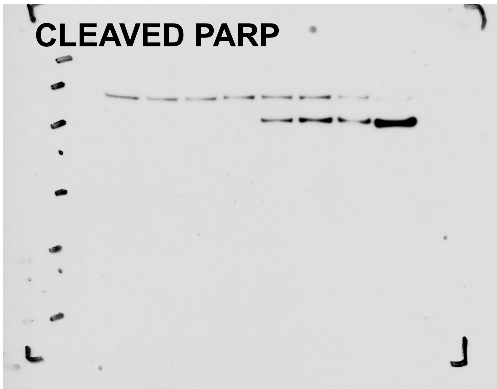

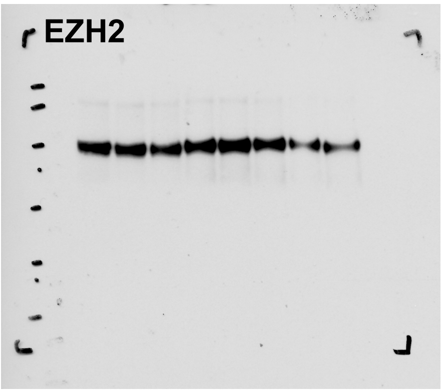

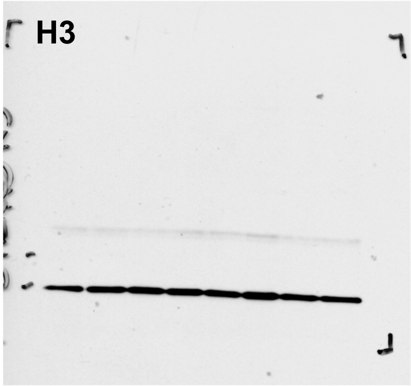

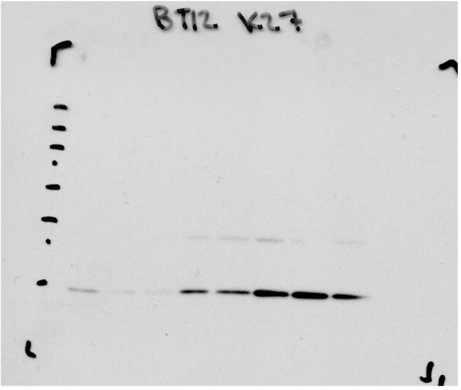

Source Data for Chasse 2020, 4G

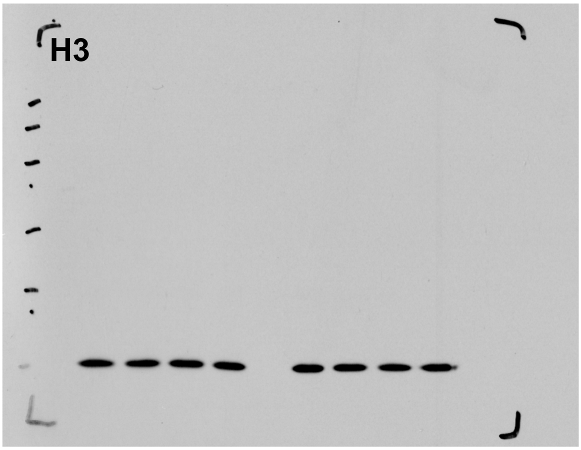

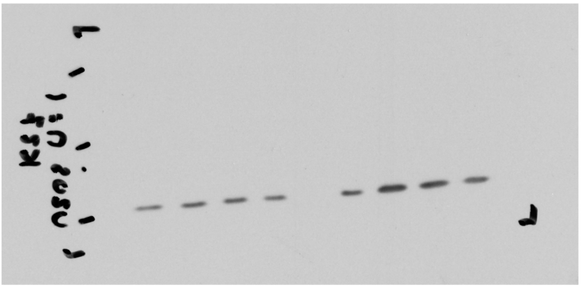

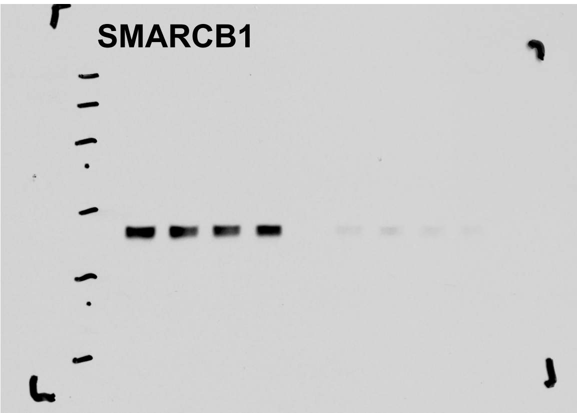

Supplement: Supplementary file 7 — Source Data for Figure 4 [file EMMM-13-e12640-s005.pdf]
